# CBX2 phase-separation contributes to homologous recombination repair and drug resistance in ovarian cancer

**DOI:** 10.1038/s41419-026-08605-4

**Published:** 2026-03-26

**Authors:** Si Sun, Lin Huang, Yujia Ma, Zheng Wei, Mengna Zhu, Mengqing Chen, Feiquan Ying, Xiaoling Zhou, Ping Yang, Yiping Wen, Qiang Yang, Liqiong Cai, Yuan Zhang, Jing Cai

**Affiliations:** 1 Department of Obstetrics and Gynecology, Union Hospital, Tongji Medical College, Huazhong University of Science and Technology, Wuhan 430022, China; 2 Department of Obstetrics and Gynecology, Peking University Third Hospital, Beijing, China; 3 Department of Obstetrics and Gynecology, Third Hospital of Shanxi Medical University, Shanxi Bethune Hospital, Shanxi Academy of Medical Sciences, Tongji Shanxi Hospital, Taiyuan, China; 4 Chinese Institutes for Medical Research, Capital Medical University, Beijing, China; 5 Department of Obstetrics and Gynecology, First Affiliated Hospital, School of Medicine, Shihezi University, Shihezi, China; 6 Department of Obstetrics and Gynecology, Xinjiang Production and Construction Corps Hospital, Urumqi, China

**Keywords:** Ovarian cancer, Ovarian cancer, Oncogenes, High-throughput screening

## Abstract

Drug resistance jeopardizes the prognosis of high-grade serous ovarian carcinoma (HGSOC) patients via DNA damage repair-coupled mechanism. The role of biomolecular phase separation in DNA damage repair has loomed. Here we find that CBX2 condensates are associated with drug resistance and contribute to DNA double-strand break (DSB) repair in HGSOC. Specifically, CBX2 condensates facilitate the recruitment of key DSB repair factors PARP1, 53BP1, and BRCA1 to chromatin. Patients with a CBX2-negative pattern exhibit the best prognosis, followed by those with non-condensate CBX2, while the worst outcomes are observed in patients with condensate CBX2. By drug screening, Ibrutinib is identified as an effective inhibitor of HGSOC cells and patient-derived organoids with CBX2 condensates. Overall, CBX2 phase separation enhances DSB repair-mediated drug resistance in HGSOC cells, and Ibrutinib may offer a viable therapeutic option for CBX2-positive HGSOC patients.

## Introduction

Epithelial ovarian cancer (EOC) is a devastating gynecological malignancy that poses a significant threat to women worldwide. High-grade serous ovarian carcinoma (HGSOC) accounts for 70–80% of EOC and has a 5 - year overall survival rate of less than 40%. The prevailing therapeutic approach for HGSOC patients encompasses surgery, succeeded by platinum-based chemotherapy. Integrating poly (ADP-ribose) polymerase inhibitor (PARPi) as maintenance therapy has been instrumental in extending the platinum-free interval, thereby yielding substantial improvements in the survival outcomes of certain patients [1]. However, approximately 20% of HGSOC patients were primarily platinum non-responders, and about 70% of platinum-sensitive cases would eventually develop platinum resistance, highlighting an imperative need for novel treatment options.

To date, a plethora of mechanisms involving platinum resistance have been proposed and elucidated, such as the decrease of intracellular drug accumulation, the sequestration of cytotoxic platinum through cellular detoxification, repair of cisplatin-induced DNA damage, attenuation of cell death, and hyper-activation of survival pathways. These findings have led to the recognition of platinum-resistant HGSOC as a spectrum of diseases characterized by CCNE1 amplification [2], homologous recombination repair (HRR) proficiency, enrichment of cancer stem cells [3], increased genomic instability, poor immune infiltration, over-expression of specific receptors, and activation of cancer-associated signaling pathways. Many pre-clinical studies and clinical trials have investigated the effectiveness of various therapeutic strategies, including immunotherapy, antibody-drug conjugates, antiangiogenic agents, ATR/WEE1 inhibitors, AKT/PI3K inhibitors and vaccines, in managing platinum-resistant HGSOC [4]. Despite the concerted efforts, the survival benefits conferred by these therapeutic approaches have been minimal.

As evidenced by laboratory and clinical observations, platinum-based chemotherapy and PARPi treatment demonstrated shared mechanisms of resistance, one of which involved DNA double-strand break (DSB) repair. DSB repair involves coordinated steps, including DNA damage sensing, transcription repression, choice of repair pathways, recruitment of repair proteins, and actual damage repair. Recent studies have highlighted the significance of phase separation, a mechanism of biomolecular interaction, in understanding the dynamic nature of DSB repair [5, 6]. Given the complexity of biomolecular phase separation, its functional implications cannot be fully comprehended through traditional molecular biology techniques such as DNA, RNA, or proteomic sequencing alone. Thus, DSB repair-associated condensates may contribute to chemotherapy resistance, which is not entirely explained by genetic or epigenetic alterations.

In addition to well-acknowledged DNA repair proteins, accumulated studies have emphasized the importance of phase separation of histone reader in DNA repair and cancer treatment. The tudor domain-containing histone reader 53BP1 plays a crucial role in forming DSB repair compartments and activating checkpoints globally through phase separation [7]. The bromodomain-containing histone reader BRD4 undergoes phase separation with Ku-80 and hinders the assembly of the non-homologous end joining (NHEJ) synaptic complex, preventing DSB loading [8]. Preclinical investigations have demonstrated that suppressing these histone readers augments the sensitivity of cancer cells to platinum-based chemotherapy and promotes synthetic lethality when combined with PARPi treatments, thereby presenting a viable and promising strategy for managing patients resistant to conventional chemotherapy [8–10].

CBX2, a chromodomain-containing histone reader, has been discovered to mediate chromatin condensation and polycomb repressive group 1 assembly through phase separation [11], and its oncogenic influences have been reported in various cancers. However, the impact of CBX2 condensates on DSB repair and chemotherapy resistance has not been fully elucidated. Here, we report that loss of CBX2 reduces DSB repair and renders HGSOC susceptibility to platinum-based treatments. Disrupting CBX2 condensates increases HGSOC’s sensitivity to platinum and PARPi in vitro and in vivo. CBX2 condensates facilitate chromatin recruitment of DSB repair proteins, promoting both NHEJ and HRR. Through drug screening, we identify Ibrutinib as a potent agent that efficiently counteracts the enhanced HRR caused by CBX2 condensates, which was further validated in patient-derived organoids (PDOs). Our findings reveal a critical role of CBX2 condensates in promoting DSB repair and demonstrate Ibrutinib as a potential treatment for platinum-resistant ovarian cancer patients.

## Results

### CBX2 confers platinum resistance to HGSOC in vitro and in vivo

First, the associations between the level of CBX2 and the clinical outcomes in EOC were analyzed based on public resources. Data from six GEO datasets demonstrated that CBX2 was elevated in EOC (GSE66957, GSE81778), serous ovarian carcinoma (GSE14407, GSE38666), and HGSOC tissues (GSE10971, GSE18520) compared to non-malignant controls (Supplementary Fig. 1a). Comparison between early- and late-stage patients from GEO dataset GSE126308 presented enrichment of CBX2 in late-stage patients (Supplementary Fig. 1b). According to ROC Plotter (https://www.rocplot.org/ovarian/index), platinum non-responders had higher level of CBX2 than responders (Supplementary Fig. 1c). In addition, survival analysis by KM-plotter suggested that high CBX2 level associated with poor progression-free survival (PFS) and overall survival (OS) in EOC patients, which was further validated with three more GEO datasets (GSE19829, GSE165808, GSE102073) (Supplementary Fig. 1d-f).

**Fig. 1:**
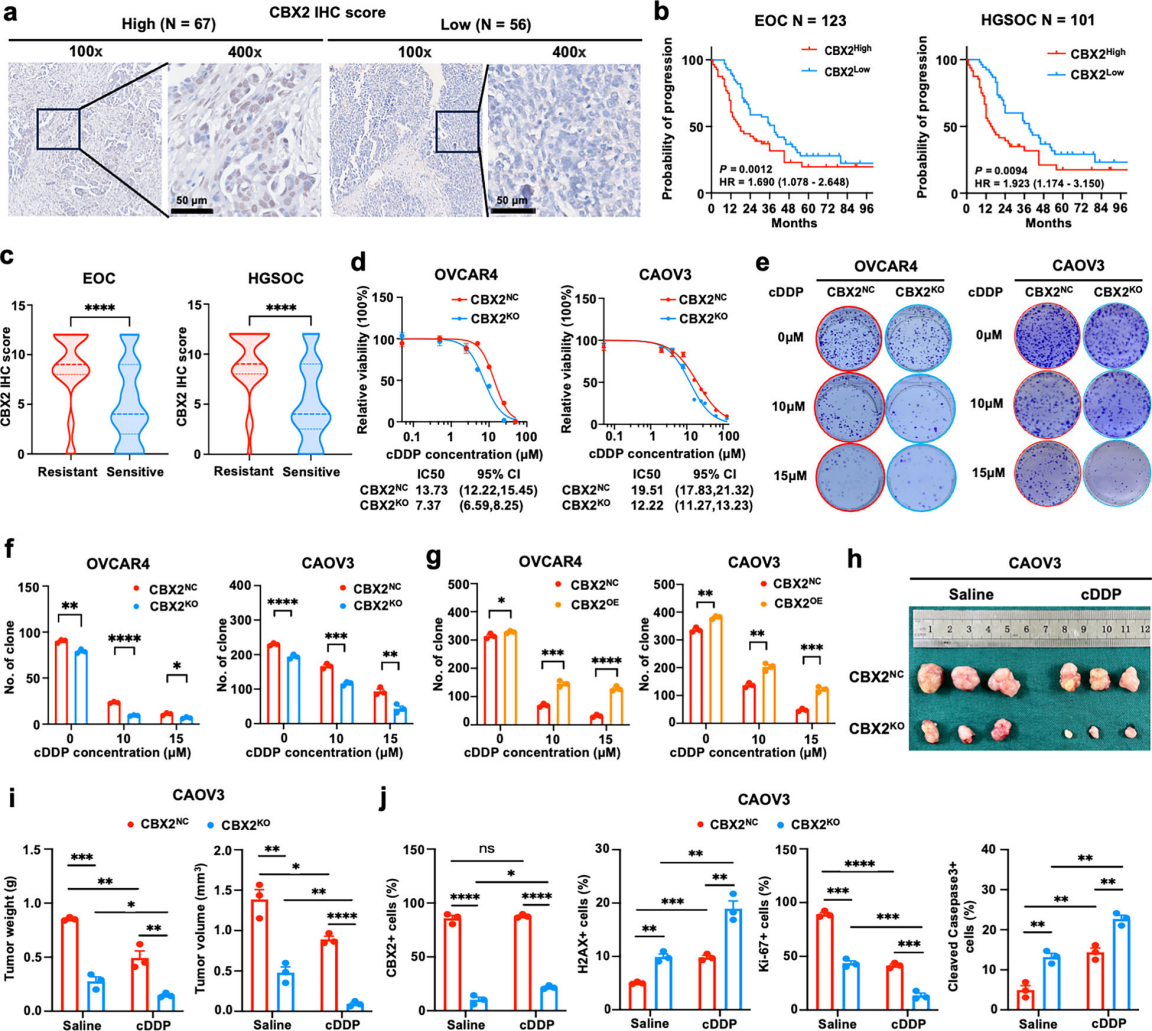
CBX2 confers platinum resistance to HGSOC. **a** Representative IHC images of CBX2 staining (nuclear, brown) from the high (*N* = 67) and the low (*N* = 56) CBX2 groups of EOC patients with a median score of 6 as the cut-off value. Scale bar, 50 μm. **b** Kaplan-Meier plots showing the progression-free survival of 123 EOC and 101 HGSOC patients with tumors expressing high and low levels of CBX2 along with hazard ratios and *P* values (high vs. low, cox regression. **c** Violin plots presenting the IHC scores of CBX2 in platinum-resistant and -sensitive EOC and HGSOC patients (Mann-Whitney test). **d** Cell viability in the CBX2 knockdown cells compared with controls after gradient concentrations of cDDP treatment for 72 h detected by MTT assay. **e** Representative images and (**f**) quantification of colony formation assays for CRISPR-Cas9 CBX2 knockout OVCAR4 and CAOV3 cells compared with controls after indicated cDDP treatment for 14 days (unpaired two-tailed Student’s t test). **g** Quantification of colony formation assay for CBX2 over-expressed OVCAR4 and CAOV3 cells compared with controls after indicated cDDP treatment for 14 days (unpaired two-tailed Student’s t test). **h** Images of subcutaneous xenografts derived from CBX2-knockout and control CAOV3 cells treated with saline and cDDP (*N* = 3). **i** Quantification of tumor volume and weight of the CBX2-knockout (CBX2^KO^) compared with the control groups (CBX2^NC^) upon treatment of cDDP (3 mg/kg, every other day) and saline on the day of sacrifice. **j** Quantification of IHC scores of CBX2, Ki-67, H2AX, and cleaved-caspase3 of tumor tissues from subcutaneous xenografts. Data represent mean ± SEM of at least three independent biological replicates. ns not significant, *P* > 0.05; * *P* < 0.05; ** *P* < 0.01; *** *P* < 0.001; **** *P* < 0.0001.

Next, the correlation between CBX2 and EOC clinical outcomes was verified through immunohistochemistry (IHC) analysis of an EOC tissue microarray (TMA) of 123 EOC tissue samples, including 101 HGSOC samples (Table. S1). EOC patients were divided into a high CBX2 group (*N* = 67) and a low CBX2 group (*N* = 56) based on the median score of IHC staining (Fig. 1a). In EOC patients, no significant difference in PFS was found between the high and low CBX2 groups. However, in HGSOC patients, those with high CBX2 IHC scores (*N* = 53) experienced worse PFS (Fig. 1b). Moreover, in both EOC and HGSOC cohorts, platinum-resistant patients had higher CBX2 IHC scores (Fig. 1c). These findings indicate a strong correlation between CBX2 and platinum responsiveness in EOC patients, especially in HGSOC patients.

We then validated whether CBX2 omitted cellular responsiveness to platinum in vitro and in vivo. First, we assessed the protein levels of CBX2 in two pairs of established drug-resistant cell lines: A2780 and its cisplatin-resistant strain, ACP, developed in vitro, as well as SKOV3 and its in vivo cisplatin-resistant strain, SKOV3-3rd, developed in vivo. The results showed that the protein levels of CBX2 were higher in the cisplatin-resistant strains (Supplementary Fig. 2a). Then, CBX2 knockout and over-expression cell models were acquired through CRISPR-Cas9 editing and lentivirus CBX2 plasmid delivery using HGSOC cell lines OVCAR4 and CAOV3 (Supplementary Fig. 2b–d). Results of MTT and cisplatin-exposure colony formation assays suggested that the reduction of CBX2 sensitized the cellular responsiveness to cisplatin (Fig. 1d-g). Likewise, CBX2 overexpression diminished the cytotoxicity of cisplatin (Fig. 1g and Supplementary Fig. 2e). We next sought to evaluate the effect of CBX2 on cellular responsiveness to cisplatin in vivo. CBX2 knockout significantly impeded tumor growth and cisplatin-induced tumor growth inhibition (TGI) by 67.6% and 70.3% in CAOV3 subcutaneous xenografts (Fig. 1h, i). IHC scores of CBX2 and Ki-67 in the CBX2-knockout group with or without cisplatin exposure were significantly lower, while IHC scores of γH2AX and cleaved caspase-3 were significantly higher (Fig. 1j). These findings align with the observed tumor growth inhibition resulting from the knockout of CBX2 (Fig. 1j and Supplementary Fig. 2f). Altogether, these findings suggest that CBX2 promotes tumor growth and confers platinum resistance to HGSOC in vitro and in vivo.

### Loss of CBX2 diminishes DNA double-strand break repair

**Fig. 2:**
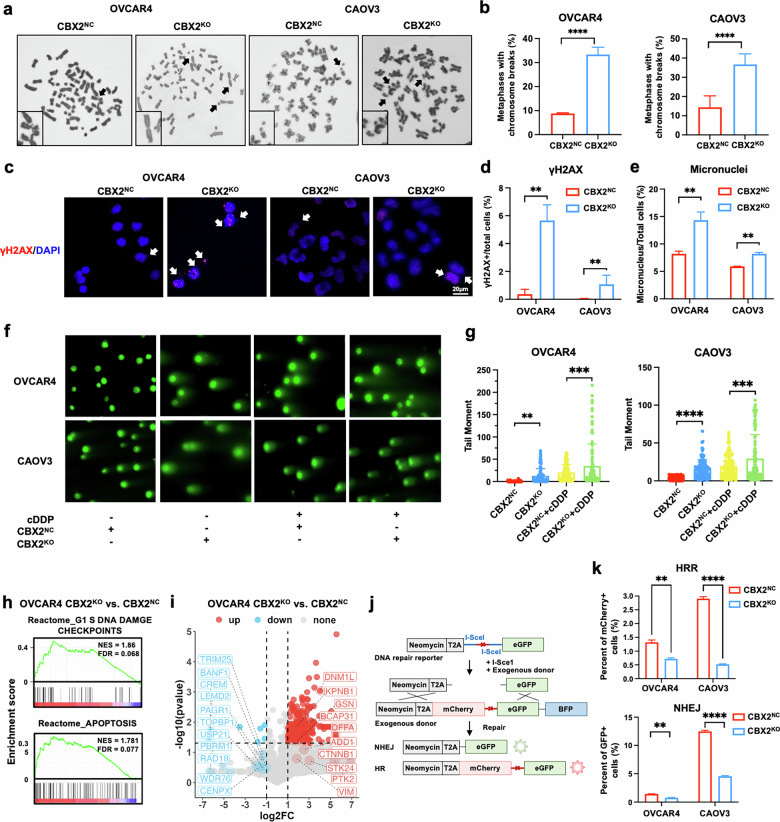
Loss of CBX2 diminishes DNA double-strand break repair. **a** Representative images of the karyotype of CBX2 knockout (CBX2^KO^) and control (CBX2^NC^) cells. **b** Quantification of chromosomal breaks in CBX2^KO^ and CBX2^NC^ cells at metaphase (unpaired two-tailed Student’s t test). **c** Representative images and quantification (**d**) of γH2AX foci and (**e**) micronuclei staining performed in the CBX2^KO^ and the control OVCAR4 cells. Scale bar, 20 μm (unpaired two-tailed Student’s t test). **f** Representative images and (**g**) quantification of the tail moment of the neutral comet assay of the CBX2^KO^ and the control OVCAR4 cells treated with 15 μM cDDP or saline for 72 h (unpaired two-tailed Student’s t test). **h** Reactome GSEA enrichment score curves of chromatin-bound proteomics from the CBX2^KO^ OVRCAR4 cells compared to the control cells. **i** Volcano plots presenting up-regulated apoptosis-associated proteins and down-regulated DNA damage checkpoints in the CBX2^KO^ compared to the control OVCAR4 cells. **j** Schematic illustration of the HR/NHEJ reporter assay. **k** Quantification of the percentage of GFP/mCherry positive cells in the CBX2^KO^ and the control OVCAR4 cells through flow cytometry (one-way ANOVA). Data represent mean ± SEM of at least three independent biological replicates. ns not significant, * *P* < 0.05; ** *P* < 0.01; *** *P* < 0.001; **** *P* < 0.0001.

A prior study discovered that the loss of CBX2 in mouse fibroblasts led to massive DSBs, chromosome instability, and notable disruption of transcripts involved in DNA repair and chromocenter formation [12]. As such, we sought to investigate whether loss of CBX2 in HGSOC cells would aggravate endogenous DSB and cisplatin-induced DNA damage. In OVCAR4 and CAOV3, CBX2 knockout increased the proportion of cells with chromosome breaks (Fig. 2a, b), γH2AX foci, and micronuclei (Fig. 2c–e). The neutral comet assay with or without cisplatin exposure suggested that CBX2 knockout led to elevated endogenous DSBs and cisplatin-induced DSBs (Fig. 2f, g). These findings suggest that CBX2 knockout leads to severe DSBs in HGSOC cells, which aligns with the prior study.

Then we intend to investigate which cluster of DNA damage repair proteins might be diminished due to the loss of CBX2. Chromatin-bound proteins from CBX2^KO^ vs. CBX2^NC^ OVCAR4 cells after cisplatin exposure were extracted, validated for component purity and CBX2 status, and submitted for proteomic analysis (Supplementary Fig. 3a–c). A total of 3318 proteins were identified. Differential protein analysis (Fold change ≥ 2, or ≤ 0.5, *P* < 0.05) showed enrichment of 401, and depletion of seven proteins in the chromatin-bound proteins of the CBX2 knockout group (Supplementary Fig. 4a). Pathway enrichment via KEGG annotation suggested that nucleotide excision repair, responsible for platinum-DNA adducts removal, was enriched in the CBX2 knockout group (Supplementary Fig. 4b). General GSEA analysis based on all detected proteins revealed enrichment of G1/S DNA damage, G2/M, and mitotic spindle checkpoints, but a marked decrease in some crucial DSB repair proteins. Meanwhile, activation of apoptosis, response to hypoxia, and the apoptotic execution phase were observed in the CBX2 knockout group (Fig. 2h, i, and Supplementary Fig. 4c). These findings prompted an investigation into whether the depletion of CBX2 exacerbated cellular apoptosis by impairing DSB repair. We evaluated the NHEJ and HR abilities in the absence of CBX2 using a previously described cell reporter assay (Fig. 2j) [13]. In CBX2-depleted cells, both NHEJ and HR were significantly reduced (Fig. 2k, Supplementary Figs. 5 and 6).

### Phase separation of CBX2 contributes to DSB repair and drug resistance of HGSOC

**Fig. 3:**
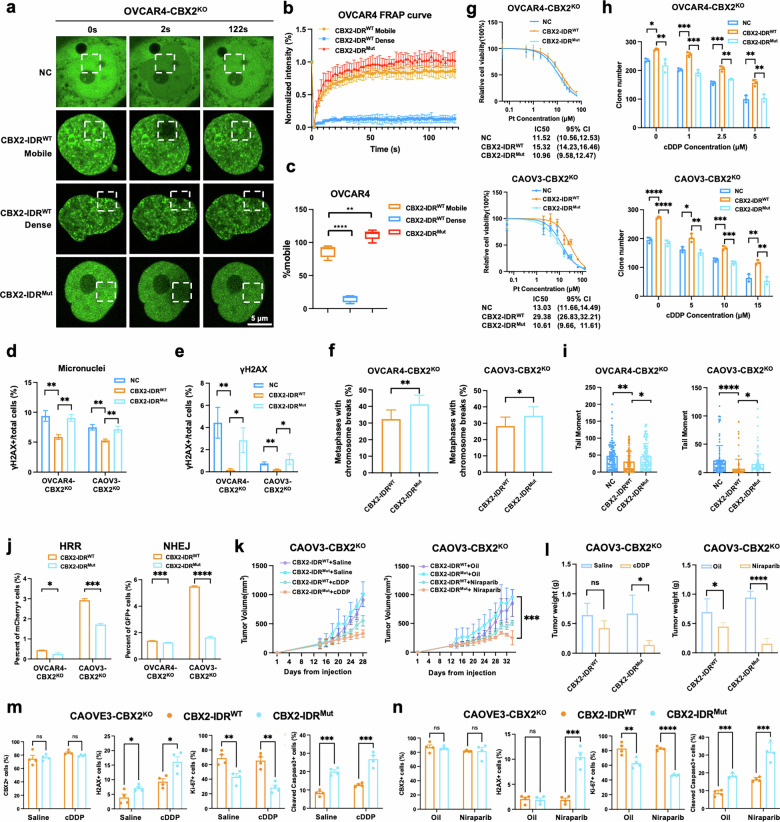
Phase separation of CBX2 contributes to DSB repair. **a** Representative confocal images of FRAP of EGFP signal in EGFP-tagged NC, CBX2-IDR^WT^, and CBX2-IDR^Mut^ transfected CBX2 knock-out OVCAR4 cells (OVCAR4-CBX2^KO^) at pre-breaching (0 seconds), breaching (2 seconds), and post-breaching (122 seconds). Scale bar, 5 μm. **b** FRAP curves of EGFP signal in EGFP-tagged CBX2-IDR^Mut^ and CBX2-IDR^WT^ cells. **c** Percentage of mobility of CBX2 in CBX2-IDR^Mut^ and CBX2-IDR^WT^ cells. **d** Quantification of γH2AX foci and (**e**) micronuclei staining performed in EGFP-tagged NC, CBX2-IDR^WT^, and CBX2-IDR^Mut^ cells. Scale bar, 20 μm (unpaired two-tailed Student’s t test). **f** Quantification of chromosomal breaks in EGFP-tagged NC, CBX2-IDR^WT^, and CBX2-IDR^Mut^ cells treated with cDDP at metaphase (one-way ANOVA). **g** Quantification of the tail moment of the neutral comet assay of EGFP-tagged NC, CBX2-IDR^WT^, and CBX2-IDR^Mut^ cells treated with 15 μM cDDP or saline for 72 h (unpaired two-tailed Student’s t test). **h** Cell viability in the EGFP-tagged NC, CBX2-IDR^WT^, and CBX2-IDR^Mut^ cells after gradient concentrations of cDDP treatment for 72 h detected by MTT assay. **i** Quantification of colony formation assay for EGFP-tagged NC, CBX2-IDR^WT^, and CBX2-IDR^Mut^ cells after indicated cDDP treatment for 14 days (unpaired two-tailed Student’s t test). **j** Quantification of the percentage of GFP/mCherry positive cells in the EGFP-tagged CBX2-IDR^WT^ and CBX2-IDR^Mut^ cells through flow cytometry (one-way ANOVA). **k** Tumor volume and (**l**) tumor weight of subcutaneous xenografts derived from EGFP-tagged CBX2-IDR^WT^ and EGFP-CBX2-IDR^Mut^ cells treated with saline, cDDP, oil and Niraparib. (**m**) and (**n**) IHC scores of CBX2, Ki-67, H2AX, and cleaved-caspase3 of tumor tissues from subcutaneous xenografts treated with saline, cDDP, oil and Niraparib. Data represent mean ± SEM of at least three independent biological replicates. ns not significant, * *P* < 0.05; ** *P* < 0.01; *** *P* < 0.001; **** *P* < 0.0001.

As CBX2 was known for its phase separation, and adequate abundance was considered a contributing factor promoting phase separation, we then set out to investigate whether CBX2 condensate contributes to HGSOC drug resistance. Since the intrinsically disordered region (IDR) was the driver of CBX2 phase separation [14, 15], we first introduced EGFP-tagged CBX2-IDR^Mut^ and CBX2-IDR^WT^ into OVCAR4-CBX2^KO^ and CAOV3-CBX2^KO^ cells to nullify CBX2’s phase separation ability as previously described (Supplementary Fig. 7a, b). Compared to the CBX2-IDR^WT^ cells, the CBX2-IDR^Mut^ failed to form condensates as expected. The fluorescence intensity of CBX2-IDR^Mut^ recovered near 100%, demonstrating ultra-high mobility. Notably, in CBX2^WT^ OVCAR4 cells, two distinct forms of condensates were identified: a mobile form exhibiting rapid recovery post-photobleaching within two minutes, and a dense form displaying loss of recovery capability (Fig. 3a–c). The fluorescence intensity recovery percentage of the mobile condensates was approximately 70%, consistent with the findings reported in previous studies [16, 17]. These results indicate successful abrogation of CBX2 phase separation by IDR mutation.

Next, we investigated whether the IDR mutation of CBX2 had any impact on DNA damage repair and cellular sensitivity to cisplatin and Niraparib in HGSOC cells. MTT and colony formation assays suggested that the introduction of EGFP-tagged CBX2^WT^ but not CBX2-IDR^Mut^ significantly increased the tolerance of OVCAR4-CBX2^KO^ and CAOVE3-CBX2^KO^ cells to cisplatin in vitro (Fig. 3d, e, Supplementary Fig. 7c). EGFP-tagged CBX2^WT^ but not CBX2-IDR^Mut^ significantly reduced the occurrence of endogenous micronuclei or DSBs and cisplatin-induced chromosome breaks or DSBs (Fig. 3d–I, Supplementary Fig. 7d–f). The DSB repair assay indicated that cells with CBX2-IDR^WT^ exhibited improved cellular NHEJ and HR abilities compared to those with CBX2-IDR^Mut^ (Fig. 3j, Supplementary Figs. 8 and 9). Notably, it was observed that the subcutaneous tumor growth rates did not exhibit a statistically significant variance between the groups of EGFP-tagged CBX2-IDR^WT^ and CBX2-IDR^Mut^. However, a marked enhancement in TGI was noted in the CBX2-IDR^Mut^ group when subjected to treatments with cisplatin and Niraparib (Fig. 3k, l, Supplementary Fig. 10a, b). IHC scores of γH2AX and cleaved-caspase 3 were significantly higher, and the IHC scores of Ki-67 were lower in the CBX2-IDR^Mut^ group after cisplatin and Niraparib exposure (Fig. 3m, n, Supplementary Fig. 10c, d). These findings suggested that CBX2 phase separation contributes to DSB repair and drug resistance.

### CBX2 promotes chromatin recruitment of key DDR factors through phase separation

**Fig. 4:**
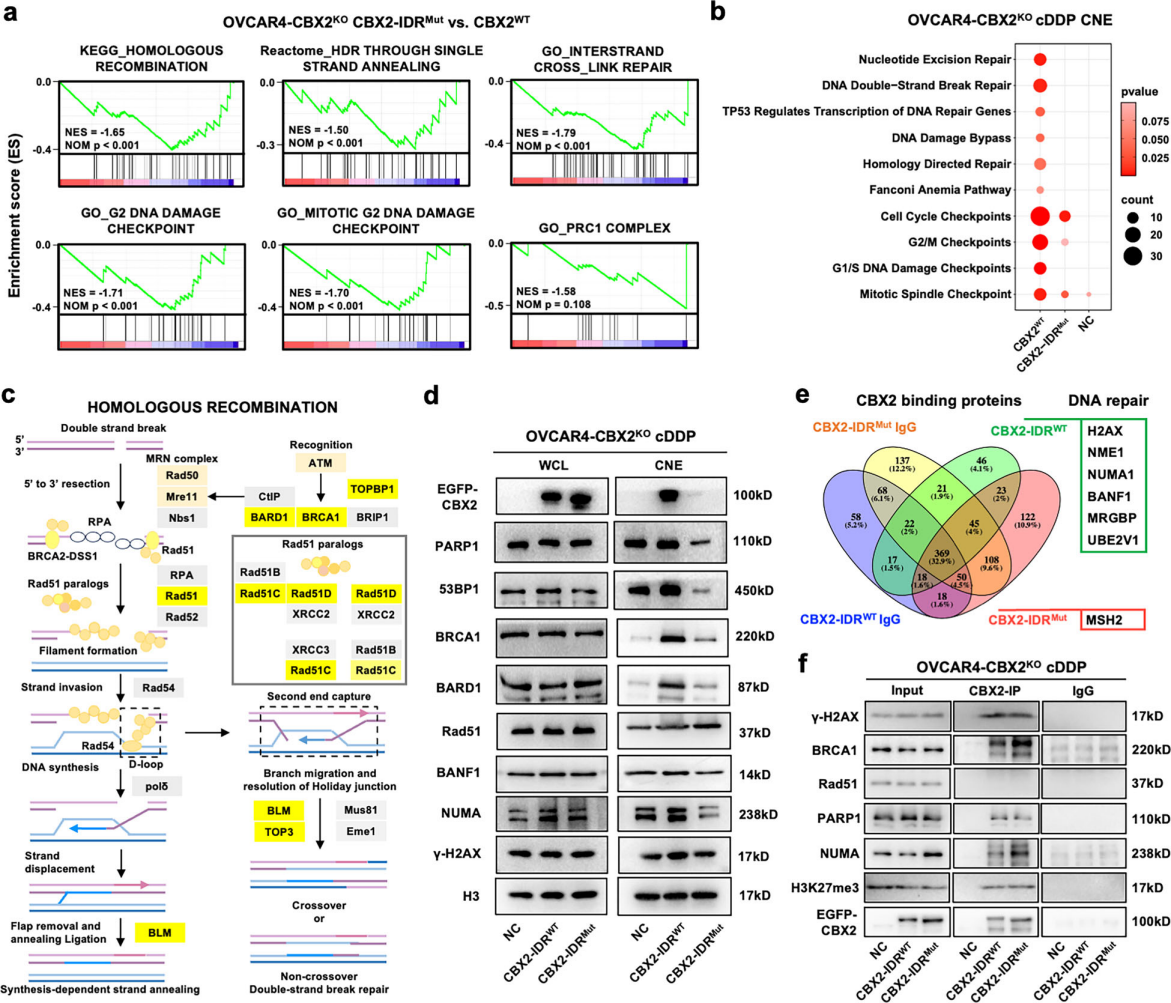
CBX2 facilitates chromatin recruitment of DDR factors through phase separation. **a** GSEA enrichment score curves of the gene expression alterations of EGFP-tagged CBX2-IDR^Mut^ compared to CBX2-IDR^WT^ cells upon treatment with 15 μM cDDP for 18 hours. ES, enrichment score; NES, normalized enrichment score; FDR, false discovery rate. **b** Bubble diagram showing changes between the chromatin-bound DDR-associated proteins among EGFP-tagged NC, CBX2-IDR^WT^, and CBX2-IDR^Mut^ OVCAR4-CBX2^KO^ cells upon treatment with 15 μM cDDP for 18 hours through proteomic analysis. CNE, chromatin-bound nuclear extract. **c** Schematic diagram presenting chromatin-bound DSB repair proteins identified in (**b**). **d** Western blots of indicated DSB proteins in the CNE and WCL of EGFP-tagged NC, CBX2-IDR^WT^, and CBX2-IDR^Mut^ OVCAR4-CBX2^KO^ cells. CNE, chromatin-bound nuclear extract; WCL, whole-cell lysate. **e** Schematic diagram presenting candidate CBX2 binding proteins detected in EGFP-tagged CBX2-IDR^WT^ and CBX2-IDR^Mut^ OVCAR4-CBX2^KO^ cells upon treatment with 15 μM cDDP for 18 hours through proteomic analysis. **f** Western blots of γH2AX, BRCA1, Rad51, PARP1, NUMA, H3K27me3, and CBX2 from lysate of input, CBX2-IP, and IgG derived from OVCAR4-CBX2^KO^ cells transfected with EGFP-tagged NC, CBX2-IDR^WT^, and CBX2-IDR^Mut^ upon treatment with 15 μM cDDP for 18 hours. CBX2-IP, lysate co-immunoprecipitated with CBX2.

Since CBX2 is a subunit of the PRC1 complex, a central transcriptional regulator that is coupled with PRC2, we first performed transcriptomic analysis to determine if the phase separation of CBX2 impacts DDR through transcriptional regulation. A total of 4 clusters of 527 differentially expressed genes between the EGFP-tagged CBX2-IDR^Mut^, CBX2-IDR^WT^, and the EGFP control groups were identified (Supplementary Fig. 11a). Several developmentally associated pathways and transcription-coupled pathways were significantly activated in the CBX2-IDR^Mut^ cells compared to the CBX2-IDR^WT^ cells (Supplementary Fig. 11b), suggesting that CBX2’s repressive functionality relies on its IDR region. Although without statistical significance, the top KEGG pathway enriched in CBX2-IDR^WT^ cells included homologous recombination and its relevant DNA repair pathways, suggesting that CBX2-IDR^WT^ but not CBX2-IDR^Mut^ contributes to global DDR activation (Fig. 4a).

Then, we postulated that phase separation of CBX2 might influence DDR at the protein level. The chromatin-bound proteomics of EGFP-tagged NC, CBX2-IDR^WT,^ and CBX2-IDR^Mut^ cells after cisplatin exposure revealed significant enrichment of DNA repair and cell-cycle checkpoints, specifically DSB repair and homology-directed repair in CBX2-IDR^WT^ cells compared to CBX2-IDR^Mut^ cells and NC cells (Fig. 4b). The primary enriched chromatin-bound DSB repair proteins in CBX2-IDR^WT^ cells included key HRR pathway components such as BRCA1, BARD1, TOPBP1, RAD51, and TOP3 (Fig. 4c). Western blot analysis was conducted to validate whether the main components of SSB and DSB repair were enriched to chromatin in CBX2-IDR^WT^ cells after exposure to cisplatin. In total cell lysate, the general protein levels of PARP1, 53BP1, BRCA1, BARD1, and Rad51 did not display significant differences between CBX2-IDR^WT^, CBX2-IDR^Mut^ and the NC cells. Chromatin-bound CBX2 was significantly more abundant in CBX2-IDR^WT^ cells compared to CBX2-IDR^Mut^ cells, which showed extremely feeble enrichment. This result suggests that although the binding of CBX2 to chromatin and histones relies on its AT hook and chromo-box, its IDR region is crucial for its enrichment on chromatin (Fig. 4d). Key DSB repair proteins 53BP1, BRCA1, Rad51, and PARP1, a protein involved in multiple DDR processes, were significantly enriched in CBX2-IDR^WT^ cells compared to CBX2-IDR^Mut^ cells (Fig. 4d), which is consistent with our chromatin-bound proteomics and our previous DDR reporter results, suggesting that CBX2-IDR^WT^ but not CBX2-IDR^Mut^ contributes to chromatin-bound DDR protein recruitment. In addition, we found that in the CBX2^KO^ control group, the enrichment levels of DNA damage repair proteins (except for HRR-related proteins) were higher than those in the mutant group and consistent with those in the wild-type group (Fig. 4d), which may be attributed to the compensatory mechanism due to complete CBX2 knock-out. These results also suggest that the CBX2-IDR^Mut^, with retained chromatin binding affinity, might exhibit non-functional occupancy, failing to execute its normal repair functions despite binding to chromatin.

Next, we investigated possible CBX2-binding proteins involved in DNA damage repair. CBX2-binding proteomic analysis suggested that CBX2-IDR^WT^, but not CBX2-IDR^Mut^, showed direct interaction with γH2AX and proteins reported to involve in DNA repair (NME1, NUMA1, BANF1, MRGBP, and UBE2V1) (Fig. 4e). Prior proteomic studies on CBX2/PRC1 interacting proteins had provided a few more CBX2-interacting candidates, including PARP1, NUMA1, and RIF1 [18]. For further validation, immunoprecipitation of CBX2 was analyzed in EGFP-tagged NC, CBX2-IDR^WT^, and CBX2-IDR^Mut^ OVCAR4-CBX2^KO^ cells after exposure to cDDP, which induces DNA damage. H3K27me3 was selected as a positive CBX2 binding control. The results demonstrated that both CBX2-IDR^WT^ and CBX2-IDR^Mut^ interacted with γH2AX, BRCA1, PAR1, and NUMA, but not Rad51, suggesting that CBX2 is involved in the initiation phase of HHR rather than the enzymatic repair phase. Additionally, the IDR mutation did not ablate the interaction between CBX2 and the DDR proteins, but affected their recruitment to chromatin (Fig. 4d, f).

### The mobile phase of CBX2 contributes to the recruitment of DDR factors

**Fig. 5:**
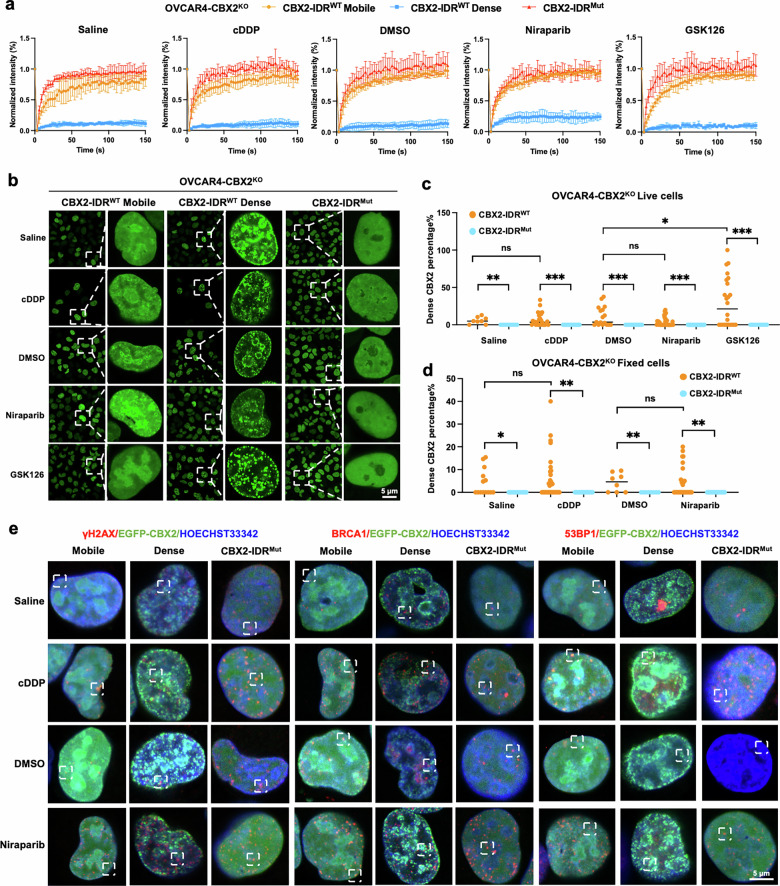
Mobile CBX2 condensates are involved in DSB repair. **a** FRAP curves of EGFP signal in EGFP-tagged CBX2-IDR^Mut^ and CBX2-IDR^WT^ OVCAR4-CBX2^KO^ cells with mobile and dense CBX2 condensates treated with saline, cDDP, DMSO, Niraparib, and GSK126. **b** Representative images of CBX2 distribution pattern in EGFP-tagged CBX2-IDR^Mut^ and CBX2-IDR^WT^ OVCAR4-CBX2^KO^ cells treated with saline, cDDP, DMSO, Niraparib, and GSK126. Scale bar, 5 μm. **c** Quantification of the percentage of dense CBX2 condensates in cells with indicated compound treatment in live and (**d**) fixed cells. **e** Representative images of γH2AX, BRCA1, and 53BP1 foci in EGFP-tagged CBX2-IDR^Mut^ and CBX2-IDR^WT^ OVCAR4-CBX2^KO^ cells treated with saline, cDDP, DMSO, and Niraparib. Scale bar, 20 μm. Data represent mean ± SEM of at least three independent biological replicates. ns not significant, *P < 0.05; **P < 0.01; ***P < 0.001; ****P < 0.0001.

During the establishment of EGFP-tagged CBX2-IDR^WT^ and CBX2-IDR^Mut^ cellular models, two forms of CBX2 condensates were identified: a rapidly recovering mobile form and a non-recovering dense form. We then inquired whether CBX2-facilitated chromatin DDR factor recruitment is subject to the form of CBX2 condensate. To ensure the ubiquity of the two forms, we first validated their presence in cells exposed to various drugs. FRAP analysis revealed that both mobile and dense forms ubiquitously existed in the EGFP-tagged CBX2-IDR^WT^ cells, but not in the CBX2-IDR^Mut^ cells (Fig. 5a and Supplementary Fig. 12a and b), suggesting that both forms rely on the IDR of CBX2. PI staining suggested that cells with dense CBX2 condensates were in a physiological state (Supplementary Fig. 12c). The two forms of CBX2 condensates displayed different patterns of intranuclear distribution. A fraction of the mobile EGFP-tagged CBX2-IDR^WT^ colocalized with chromatin, with the remainder dispersed throughout the nucleus. Conversely, the dense EGFP-tagged CBX2-IDR^WT^ condensates were exclusively associated with chromatin without any evidence of dispersion within the nuclear space (Supplementary Fig. S13a, b). In conjunction with FRAP analyses, the chromatin-associating mobile CBX2 condensates exhibited a dynamic interaction with chromatin. At the same time, the non-recovering dense CBX2 appeared entangled within the chromatin structure, transitioning into a solid phase.

We then evaluated whether the percentages of the two forms of CBX2-IDR^WT^ would change upon exposure to cisplatin and Niraparib in both live (Fig. 5b, c) and fixed cells (Fig. 5d, e). Cells treated with cisplatin and Niraparib exhibited no significant difference in non-recovering dense CBX2 condensates compared to controls treated with saline and DMSO in both live and fixed cells (3.92% to 12.17%). However, when live cells were treated with GSK126, an H3K27me3 inhibitor, the percentages of cells with non-recovering dense CBX2 condensates increased (Fig. 5b–e), suggesting that immobile CBX2 condensation was not due to CBX2-H3K27me3 interaction but rather likely through the AT-hook [11].

Since we had validated the direct interaction between CBX2 and γH2AX, we then sought to determine which form of CBX2 was involved. Immunofluorescence assays indicated that CBX2-IDRMut exhibited a uniform distribution in the nuclei, as expected. Upon treatment with cisplatin and Niraparib, though most of the CBX2-IDR^WT^ dispersed across the nuclei, a small fraction formed condensates that colocalized with γH2AX foci. In contrast, the non-recovering dense CBX2-IDR^WT^ and γH2AX foci exhibited complete exclusivity (Fig. 5e, Supplementary Fig. S14a). The distribution of mobile CBX2 condensates displayed a similar colocalization pattern with 53BP1 and BRCA1 foci, while the dense CBX2 condensates displayed complete exclusivity with the foci of 53BP1 and BRCA1 (Fig. 5e, Supplementary Fig. S14b, c).

Further, the expression levels and the functionality of the reconstituted EGFP-tagged CBX2^WT^ were examined between the parental OVCAR4 and the reconstituted EGFP-tagged CBX2^WT^. The expression level of CBX2 in the reconstituted EGFP-tagged CBX2^WT^ was slightly higher than that in the parental cells, presenting similar IC50 by MTT and a slight increase in resistance to higher doses of cDDP as indicated by the colony formation assay, which was in line with previous results in this study (Supplementary Fig. 14a–d). The addition of the N-terminal EGFP tag did not change the subcellular location of CBX2 (Fig. 3a), nor did it affect CBX2’s binding affinity to H3K27me3 near the N-terminal region or its role in drug resistance (Fig. 4f, Supplementary Fig. 15a–d). The EGFP-tagged CBX2^WT^ effectively demonstrated the drug-resistant characteristics of the CBX2 protein, serving as a reliable alternative for research.

### HGSOC cells with CBX2 condensates were sensitive to Ibrutinib

**Fig. 6:**
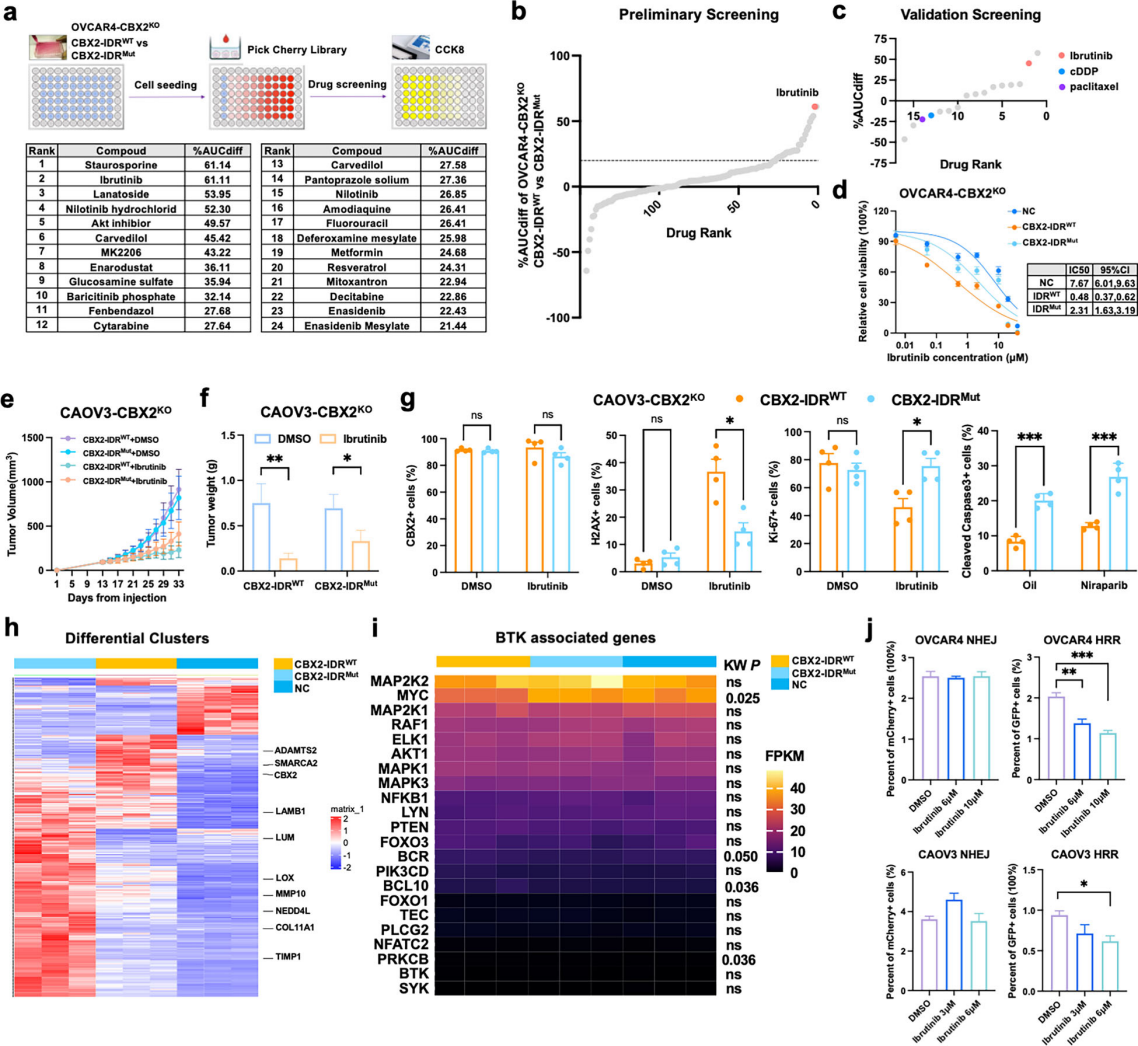
Ibrutinib exhibits anti-tumor effects on tumors containing CBX2 condensates. **a** Schematic illustration of the compound library screening and the rank of the compound with a percent difference in area under curve values (%AUC_Diff_) in EGFP-tagged CBX2-IDR^WT^ cells compared to CBX2-IDR^Mut^ cells, more than 20% in the initial screen. **b** Waterfall plots of all the drugs ranked in the initial screen and (**c**) the validation screen. **d** Cell viability of EGFP-tagged NC, CBX2-IDR^WT^, and CBX2-IDR^Mut^ OVCAR-CBX2^KO^ cells treated with gradient concentrations of Ibrutinib for 72 h evaluated by MTT assay. (**e**) Tumor volume and (**f**) tumor weight of subcutaneous xenografts derived from EGFP-tagged CBX2-IDR^WT^ and CBX2-IDR^Mut^ CAOV3-CBX2^KO^ cells treated with DMSO and Ibrutinib. **g** IHC scores of CBX2, Ki-67, H2AX, and cleaved-caspase3 of tumor tissues from subcutaneous xenografts. **h** Heatmap showing differential gene expression and (**i**) BTK-associated gene expression changes between the EGFP-tagged NC, CBX2-IDR^WT^, and CBX2-IDR^Mut^ cells. **j** Quantification of the percentage of GFP/mCherry positive cells in parental OVCAR4 and CAOV3 cells transfected with the HR/NHEJ reporter system, exposed to the indicated concentrations of Ibrutinib by flow cytometry (one-way ANOVA). Data represent mean ± SEM of at least three independent biological replicates. ns not significant, *P < 0.05; **P < 0.01; ***P < 0.001.

To identify a pharmacological agent capable of selectively targeting cells with CBX2 condensates, we conducted drug screening using EGFP-tagged CBX2-IDR^WT^ and CBX2-IDR^Mut^ OVCAR4-CBX2^KO^ cells with the pick-cherry compound library. Preliminary screening identified 24 compounds with a percent of difference of area under the curve (%AUCdiff) of more than 20% that might have more potent cytotoxicity to the CBX2-IDR^WT^ phenotype (Fig. 6a, b). Subsequent validation screening with a more comprehensive drug range was performed on the top 14 compounds from preliminary screening, cDDP, and paclitaxel (Fig. 6c). Compounds displaying > 20%AUC_Diff_ values from the validation screening were further validated through the MTT assay. The results showed that Ibrutinib exhibited the most significant cytotoxicity to cells with CBX2 phase separation ability (Fig. 6d, Supplementary Fig. 16a). Further, Ibrutinib remained cytotoxic to EGFP-tagged CBX2^WT^ cells in vivo (Fig. 6e–g, Supplementary Fig. 16b and c).

We then investigated the possible explanation for Ibrutinib’s efficacy against EGFP-tagged CBX2-IDR^WT^ cells compared to its ineffectiveness in CBX2-IDR^Mut^ cells. First, we investigated whether Ibrutinib interfered with CBX2 phase separation. FRAP assay results demonstrated that, similar to patterns observed with DMSO treatment, both mobile and dense CBX2 condensates were observed upon Ibrutinib exposure, while CBX2-IDR^Mut^ maintained a liquid-like state (Supplementary Fig. 17a, b). The percentage of cells with non-recovering dense CBX2 condensates showed no significant difference between the groups treated with Ibrutinib and DMSO (Supplementary Fig. S17c, d). These results suggested that Ibrutinib did not interfere with the formation of CBX2 condensates. Given that Ibrutinib functions as an inhibitor of Bruton’s tyrosine kinase (BTK), we next explored whether impaired phase separation of CBX2 might cause dysregulation of BTK-associated genes. Based on the mRNA sequencing data of EGFP-tagged CBX2-IDR^Mut^ compared with CBX2-IDR^WT^ cells, differential clusters analysis and pathway enrichment analysis indicated that extracellular matrix organization genes were significantly impacted by CBX2 phase separation (Fig. 6h). BTK-associated genes displayed no statistical mRNA level difference with significant numerical variation between CBX2-IDR^WT^ and CBX2-IDR^Mut^ cells except for MYC, which was a known CBX2 target (Fig. 6i). Since a few studies have reported that Ibrutinib could inhibit DNA damage repair, especially the principal factors involved in HRR [19], we tested whether Ibrutinib inhibited DSB repair in HGSOC cell lines. According to the DSB repair reporter assay, Ibrutinib significantly impaired cellular HRR but not NHEJ (Fig. 6j). Therefore, we speculated that Ibrutinib detrimentally affects the HRR mechanisms upon which CBX2-IDR^WT^ cells depend on surviving, resulting in cytotoxic effects on these cells.

### CBX2 pattern predicts survival of HGSOC patients and sensitivity to Ibrutinib

**Fig. 7:**
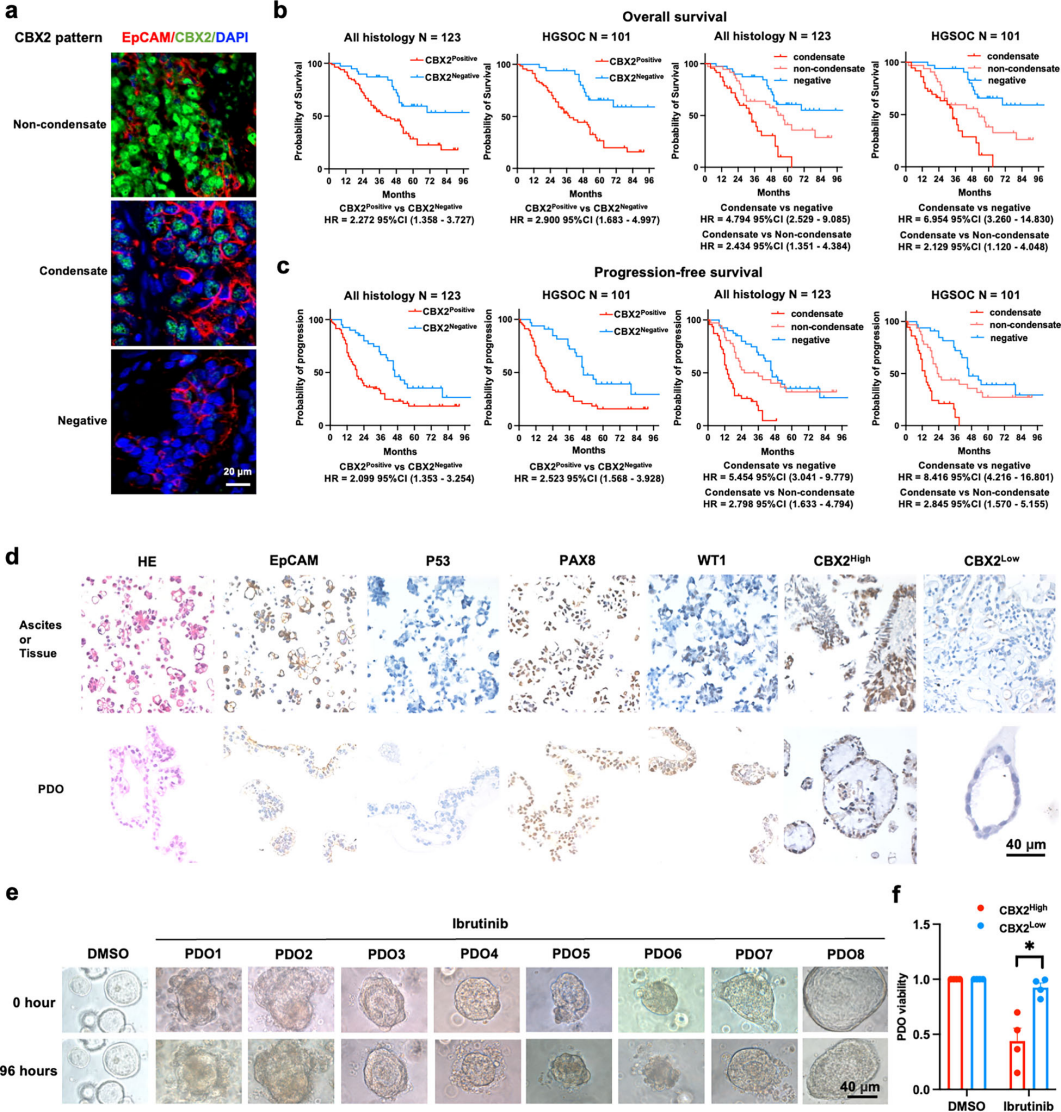
CBX2 predicts survival of HGSOC patients and sensitivity to Ibrutinib. **a** Representative images of patients with non-condensate, condensate, and negative CBX2 patterns. Red, EpCAM; Green, CBX2; Blue, DAPI. Scale bar, 20 μm. **b** The Kaplan-Meier plot depicts the overall survival, and (**c**) progression-free survival of patients with all histologies and those with HGSOC presenting varying CBX2 positivity and patterns, and the corresponding hazard ratios (log-rank test, cox-regression). **d** Representative images of IHC staining of EpCAM, P53, PAX8, WT, and CBX2 of PDOs and their respective tissues of origin. Scale bar, 40 μm. **e** Representative images of PDOs derived from 8 patients before and after treatment with DMSO and Ibrutinib for 96 hours. Scale bar, 40 μm. **f** Viability of PDOs with high and low IHC score of CBX2 treated with DMSO and Ibrutinib for 96 h. PDO, patient-derived organoid (unpaired two-tailed Student’s t test). Data represent mean ± SEM of at least three independent biological replicates. ns not significant, *P < 0.05.

Based on the evidence presented, the efficiency of DSB repair and the sensitivity of HGSOC cells to platinum and PARP inhibitors were found to be closely linked to CBX2 and its different forms of condensates. Consequently, we investigated whether the survival of patients was associated with different patterns of CBX2 condensates. Tissue immunofluorescence (IF) demonstrated three patterns of CBX2: condensate, non-condensate, and negative (Fig. 7a). A positive CBX2 IF signal and a condensate CBX2 IF pattern were significantly associated with platinum resistance in both cohorts of ovarian cancer of all histologies (*N* = 123) and HGSOC (*N* = 101) (Tables S2 and S3). Tissue samples from the resistant group presented a significantly higher fraction of CBX2 IF-positive cells and CBX2 condensates (Supplementary Fig. 18a–d). Patients with positive CBX2 IF signals generally had worse OS and PFS in cohorts of all histology (*N* = 123) and HGSOC (*N* = 101) (Fig. 7b, c). Moreover, patients showing a pattern of condensate CBX2 had the worst OS and PFS. Individuals with a pattern of non-condensate CBX2 exhibited intermediate levels of OS and PFS, while patients with a negative CBX2 pattern had the best OS and PFS (Fig. 7b, c). These findings highlight the prognostic significance of CBX2 condensate patterns in the clinical outcomes of patients.

We then assessed the toxicity of Ibrutinib on HGSOC cells with different CBX2 levels. Patient-derived organoids (PDOs) were cultured from ascites or tissue samples obtained from eight HGSOC patients. The representativeness of PDOs was confirmed through IHC analysis of EpCAM, P53, PAX8, and WT1, as compared to their sources (Fig. 7d). The PDOs were categorized into CBX2^High^ and CBX2^Low^ groups based on their CBX2 IHC scores. They were then subjected to treatment with Ibrutinib for 96 h (Fig. 7d, e). The PDOs with higher CBX2 IHC scores exhibited a notable sensitivity to Ibrutinib. These results suggest that CBX2 levels may be a potential indicator for predicting Ibrutinib sensitivity.

## Discussion

The field of phase separation of in vivo cellular components is emerging, with key questions still under investigation. In vivo phase-separation and relevant biological effects, associations with clinical diseases, and potential therapeutic targets are still under active research. Previous studies had clarified how IDR mediates CBX2 phase separation, and the regulating role of total CBX2 in genomic instability and cellular stemness [12, 15, 16, 20]. However, the biological effects of CBX2 phase separation remain unclear, as does its potential association with clinical diseases or its potential as a therapeutic target. This study investigated how CBX2 phase separation enhances DSB repair and contributes to platinum resistance in HGSOC. Our results also indicate that Ibrutinib exhibits specific anti-tumor effects on tumor cells with CBX2 condensates, suggesting its potential clinical application for treating platinum-resistant HGSOC patients. Our research highlights a novel mechanism underlying drug resistance involving CBX2 phase separation and identifies a new drug candidate for resistant HGSOC models with potential for clinical translation.

In addition to previous controversial observations on whether CBX2 is involved in DSB repair [21, 22], our study provides evidence of the recruitment of CBX2 to DSB foci alongside enhanced DSB repair efficiency in HGSOC dependent on CBX2 phase separation. DSB repair is a complex process that involves DSB sensing, DNA damage response, repair pathway choice, and DSB repair. Here, we observed that upon the comparable dosage of cisplatin exposure, CBX2 phase separation abrogation caused impairment of chromatin recruitment of a wide array of DSB repair-associated proteins, including DSB sensor PARP1, pathway choice mediators 53BP1 and BRCA1, end resection/recombination core protein Rad51 but not γH2AX, which is a crucial docking site for DSB repair proteins promoted by MRN/ATM signaling [23]. Regarding DSB repair, PARP1 is a crucial DSB sensor in the S and G2 phases, promoting HR repair and alt-NHEJ over c-NHEJ [24]. 53BP1 and BRCA1 are two fundamental pathway choice regulators competing for docking at H2AK15Ub to direct NHEJ or HR [25]. Our findings suggest that the disruption of CBX2 phase separation did not interfere with the formation of γH2AX but did inhibit the chromatin recruitment of PARP1, 53BP1, BRCA1, BARD1, and RAD51. Simultaneously, CBX2 condensates appeared to contribute as a scaffold for enlisting general DSB repair proteins, particularly those engaged in HR repair, since disrupting CBX2 phase separation hindered both HR and NHEJ and significantly impeded the chromatin recruitment of crucial proteins involved in each phase of DSB repair, particularly HR repair.

According to recent discoveries, it seems that nearly every key link of DDR pathways, including DSB synapsis, DSB initiation, repair pathway choice, and DSB repair, was supported by inter-molecular phase separation to fulfill physical foci formation, self-functionality, and downstream DSB factor recruitment. Namely, PARP1 condensates drive the formation of DSB foci, mediates DNA synapsis at DSB sites, and recruits FUS [5]. FUS undergoes phase separation, promotes proper arrangement of γH2AX, initiates DDR, and recruits 53BP1. 53BP1 forms condensate with MDC1 to propagate Ku70 and Ku80 assembly at DSB sites [26]. MRNIP phase separation concentrates DSB-bound DSB sensors MRNs and promotes ATM autophosphorylation [27]. RNF168, responsible for H2AK15Ub and subsequent recruitment of either 53BP1 or BRCA1, was restricted to DSB sites by SUMOylation-regulated phase separation. RAP80, a component of the BRCA1-A complex, directed DSB recruitment of BRCA1 through phase separation [28]. TOPBP1, a crucial BRCA1 associating protein, forms condensate to switch on the ATR signaling [29]. Although the detailed process of how phase separation regulates and propels global DDR has not been completely elucidated, it is evident that phase separation among principal DSB regulators significantly contributes to the facilitation and advancement of the DSB repair mechanism. Our findings here have unveiled that CBX2 serves as a novel key joint within the network of phase separation control mechanisms integral to the DDR.

In this study, the CBX2-DSB repair protein interaction was only observed when CBX2 was in its mobile form, while the immobile CBX2 condensates demonstrated complete exclusive association with DSB repair condensates. Although we did not figure out the physiological function of immobile CBX2, our results suggested that it was a physical state. Notably, the characteristics of the immobile CBX2 condensates observed in this study were consistent with previous report [11]. We believe that the formation of immobile CBX2 requires both IDR region and AT region since CBX2-IDR^mut^ in this study and CBX2^AT-P2A^ reported by Kent et al. demonstrated similar liquid-like distribution. Additionally, the pattern and the distribution of immobile CBX2 condensates observed here resembled those formed by CBX2 and chromatin when RING1A/B and BMI1/MEL18 were depleted [11]. It was highly likely that the immobile CBX2 condensates resulted from CBX2 disassembled from PRC1 and stabilized on chromatin through its AT-hook. Based on the findings by Brown et al., condensates with mobile CBX2 represent PRC1 condensate scaffolded by CBX2 [11]. Therefore, the co-localization of mobile CBX2 condensates and DSB repair proteins here might actually represent the dynamic interaction of a more complex system. Considering that several CBX2 clients were involved in DSB regulation, it is worth further investigating whether CBX2 phase separation provides a conformational basis for the DSB repair regulatory activity of PRC1, which would add a broader understanding of global DDR.

Ibrutinib is an orally administered small molecular BTK inhibitor approved by the FDA for treating hematologic malignancies. Its off-target activity against tyrosine kinases that harbor homologous cysteines to BTK, including EGFR, HER2, and HER4, has prompted research on applying Ibrutinib in solid tumors, especially breast cancers [30]. Perhaps due to the prevalent absence of the molecular phenotype conducive to Ibrutinib therapy, there has been a limited exploration into the effectiveness of Ibrutinib in the treatment of ovarian cancer. Here, the identification of Ibrutinib as a potent agent against cells exhibiting CBX2 condensates was a serendipitous outcome from screening a comprehensive compound library. Despite not finding any known BTK target alterations, we hypothesized that Ibrutinib disrupts the HRR mechanisms necessary for the survival of EGFP-tagged CBX2-IDR^WT^ cells, leading to their cytotoxic effects. Interestingly, a recent study documented the use of Ibrutinib in treating a patient with late-stage platinum-resistant low-grade serous ovarian cancer. Ibrutinib and related drugs were identified through drug screening based on PDOs, and the patient experienced over 24 months of stable disease following treatment despite not having the identified alterations that could explain BTK or EGFR inhibitor sensitivity. The study also alluded to as-yet-unpublished data suggesting that ibrutinib may have clinical benefits for over 10% of ovarian cancer patients, including those with HGSOC [31]. Taken together, Ibrutinib seems to be a promising candidate treatment for platinum-resistant ovarian cancer patients with more refined molecular typing and a basis for patient selection.

In summary, our results demonstrate that CBX2 condensates contribute to drug resistance by promoting DSB repair in HGSOC. Ibrutinib demonstrates an exceptional anti-tumor effect on ovarian cancer with CBX2 condensates. Our findings provide a rationale for the application of Ibrutinib for platinum-resistant ovarian cancer patients with CBX2 condensates.

## Materials and Methods

### Cell lines, cultures, and transfections

Human ovarian cancer cell lines OVCAR4 (RRID:CVCL_1627) and CAOV3 (RRID:CVCL_0201) were purchased from the China Center for Type Culture Collection, Wuhan, China. Cells were maintained in Dulbecco’s modified Eagle’s/F12 medium (DMEM/F12, L310KJ) supplemented with 10% FBS (Biochannel, BC-SE-FBS01) and cultured in standard conditions 37 °C and 5% CO2. All human cell lines were authenticated by short tandem repeat testing and were verified to be mycoplasma contamination-free.

CBX2^KO^ cells of OVCAR4 and CAOV3 were obtained using the CRISPR-Cas9 system in this study. The sgRNA sequences targeting CBX2 (Sg859 5’-3’ ACCAGCCGCGCCACTTGACC, Sg861 5’-3’ GAGCTTGGAGCGCCGGCTGC) were synthesized by GeneChem (Shanghai, China). Cells undergo 7 days of G418 selection for stable CBX2^KO^ phenotype. Lentivirus-directed EGFP-CBX2-IDR^WT^ and EGFP-CBX2-IDR^Mut^ transfection was performed in CBX2^KO^ cells of OVCAR4 and CAOV3. EGFP-CBX2-IDR^WT^ and EGFP-CBX2-IDR^Mut^ were synthesized by GeneChem (Shanghai, China).

### Animals

Four to five-week-old female BALB/c nude mice were acquired from Charles River Laboratories (Beijing, China). The mice were kept in specific pathogen-free (SPF) conditions with controlled temperature (22–26 °C), humidity, and a 12-hour light/dark cycle, and were randomly assigned to experimental groups. All animal experiments were conducted in strict accordance with the People’s Republic of China Legislation Regarding the Use and Care of Laboratory Animals. The number of animals used was the minimum required to reliably assess the primary endpoints in accordance with the principle of reduction. No blinding was performed for animal treatment, data collection, or data analysis. The protocols used in this study were approved by the Institutional Animal Care and Use Committee at Tongji Medical College, Huazhong University of Science and Technology (Approval Number: 20220824001).

### Patient-derived organoids establishment and culture

The establishment of organoids in this study adhered to the methodologies established by Sarah J. Hill et al. [32]. Briefly, ovarian cancer specimens were freshly obtained, dissected into 1 mm3 fragments, digested at 37 °C for 30 min (2.0 mg/mL mixture of collagenase I, II, and IV), and centrifugated to remove supernatant. The remains were resuspended in a basal medium and passed through a 70 μm filter (BD Biosciences) to achieve a uniform cell suspension. This suspension was subjected to a further centrifugation step and subsequently washed with Advanced DMEM/F12 (Gibco, 12634010) supplemented with 1% penicillin-streptomycin (BasalMedia, S110JV), 1X Glutamax (Gibco, 35050061), and 1% HEPES (stemcell, 07200). The cell suspension was mixed with Matrigel in a 1:3 ratio (Corning, Catalog number CB-40230C). Aliquots of 15 μL were pipetted into 96-well plates and incubated at 37 °C. Immediately after the consolidation of matrix gell, 100 μL of culture medium was supplemented to each well.

The culture medium comprised advanced DMEM/F12 enriched with 1% penicillin-streptomycin, 1X Glutamax, 1% HEPES, and a cocktail of growth factors and supplements including 100 ng/mL R-spondin 1 (Peprotech, catalog number 120–38), 100 ng/mL Noggin (Peprotech, catalog number 120–10 C), 50 ng/mL EGF (Peprotech, catalog number 100–15), 10 ng/mL FGF-10 and FGF2 (Peprotech, catalog numbers 100–26 and 100–18B respectively), 1× B27 (Stemcell, 05731), 10 mM Nicotinamide (Sigma Aldrich, Catalog Number N0636), 1.25 mM N-acetylcysteine (Sigma Aldrich, catalog number A9165), 1 μM Prostaglandin E2 (R&D Systems, catalog number 2296), 10 μM SB202190 (Sigma Aldrich, catalog number S7076), and 500 nm A83–01 (Sigma Aldrich, catalog number SML0788), alongside Y-27632 dihydrochloride (Stemcell, 72302) to support optimal organoid growth and differentiation.

The procedures were rigorously conducted in accordance with the ethical standards delineated in the Declaration of Helsinki. The experimental protocols were ethically sanctioned by the Research Ethics Committee of Union Hospital, affiliated with Tongji Medical College, Huazhong University of Science and Technology, Wuhan, China.

### Ovarian cancer tissues

Tissue samples from a total of 123 patients were obtained from ovarian cancer patients admitted to Union Hospital, Tongji Medical College, Huazhong University of Science and Technology between August 2008 and October 2022. 123 non-consecutive, unselected primary ovarian cancer specimens were included in the tissue microarray. The tumor samples were collected within one hour after resection from the primary site. Formalin-fixed paraffin-embedded (FFPE) tissue blocks were prepared according to the standard procedure. Tissue cylinders of 2 mm in diameter were punched from representative areas of each block with regard to the matching H&E staining control by a MiniCore Control Station (Alphelys Sarl, France). The Selected tissue cylinders were re-arranged and brought into three paraffin blocks by a semi-automated tissue arrayer (Beecher Instruments, Sun Prairie, WI, USA). 4 μm section slides were prepared for further use. The study protocol was approved by the Independence Ethics Committee of Union Hospital, Tongji Medical College, Huazhong University of Science and Technology (20210567). Informed consent was obtained in accordance with the protocol.

### Immunohistochemistry

Briefly, antigen retrieval was performed by boiling the slides in citrate buffer (10 mM, pH 6.0) after deparaffinization and rehydration. After slides were rinsed in PBS and blocked for 30 min with 5% bovine serum albumin (BSA). Slides were incubated overnight at 4 °C with primary antibodies. Slides were washed thrice with PBS and then incubated with the HRP-labeled secondary antibody for 1 h at room temperature. Subsequently, a two-step detection kit (ZSGB- BIO, SP-9001, and SP-9002) was used for IHC and hematoxylin for nuclear staining. After mounting, slides were scanned by Vectra Polaris (Perkin Elmer). Primary antibody dilutions were as follows: mouse anti-CBX2 (Cat#: MA5-38465, 1:200, Invitrogen), rabbit anti-Ki-67 (Cat#: 30-9, 0.2 μg, Ventana), mouse anti-p16 (Cat#: E6H4, 0.1 μg, Ventana), H2AX, BRCA1, 53BP1, RAD51.

### Immunohistochemistry imaging and scoring

The stained slides of ovarian cancer tissue samples were scanned by Pannoramic MIDI (3DHISTECH, Hungary) and evaluated by two pathologists independently. The IHC scoring of CBX2 was determined based on the percentage of positive cells (0, 0–10%; 1, 10–25%; 2, 25–50%; 50–75%; 4, 75%–100%) and the intensity of staining (0, negative; 1, faint; 2, moderate; 3, strong). The IHC score for CBX2 was calculated by multiplying the score for the percentage of positive cells by the score for staining intensity, resulting in possible scores of 0, 1, 2, 3, 4, 6, 8, 9, and 12. The patients were classified into the CBX2^High^ group (*N* = 67) and the CBX2^Low^ group (*N* = 56) based on a cut-off value of 6 (median).

The xenograft samples were prepared as stained slides and subsequently analyzed using an Olympus microscope for photographic documentation. IHC staining was performed to evaluate the expression levels of CBX2, γH2AX, Ki-67, and cleaved-caspase 3. The proportion of positively stained tumor cells was quantified using ImageJ software, based on five random fields of view at an objective magnification of 40x.

### MTT assay

MTT assay (Selleck) was employed To assess cell viability in response to varying drug concentrations. Briefly, 5000 cells were plated in 96-well plates (Corning). The following day, cells were treated with the indicated concentrations of drugs for 72 h. Subsequently, cell viability was determined by MTT according to the manufacturer’s instructions. The absorbance readings were taken using a Thermo plate reader. Background values from empty wells were subtracted, and data were normalized to vehicle-treated control.

### Colony formation assay

One thousand cells were seeded per well into six-well plates and cultured with medium supplemented with different concentrations of cisplatin for two weeks until colonies emerged. Colonies with more than 50 cells per colony were counted using Fiji after being fixed with 4% formaldehyde for 30 min and stained with 0.1% crystal violet overnight. The experiments were performed in at least three biological replicates.

### Apoptosis analysis

Indicated cells (2 × 10^5^) cells were seeded in 6-well plates per well and treated with the indicated compounds for 72 hours. Apoptosis was assessed using the PE Annexin V Apoptosis Detection Kit I (BD Biosciences 559763 or 556547) according to the manufacturer’s instructions. Flow cytometry analysis was conducted on an ID7000 Spectral Cell Analyzer (Sony Biotechnology).

### Western blot

Cells were harvested and lysed using the Mammalian Cell & Tissue Extraction Kit (BioVision, K269), along with protease inhibitors (Roche, 11873580001) and a phosphatase inhibitor cocktail (Bimake, B15001). The protein concentration was determined using the Quick Start Bradford 1× Dye Reagent (Bio-Rad, 5000205). Subsequently, the cell lysates were separated on SDS-PAGE gels and transferred onto 0.45 or 0.2 μm PVDF membranes (Millipore, IPVH00010, or ISEQ00010). The blots were then blocked in 5% milk in TBST (TBS/0.1% Tween-20) and incubated with primary antibodies at 4 °C overnight. Primary antibody dilutions were as follows: rabbit anti-CBX2 (Cat# 15579-1-AP, 1:1000, Proteintech, RRID:AB_2737362), mouse anti-Cas9 (Cat#: 14697, 1:1000, Cell Signaling Technology), rabbit anti-AKT (Cat#: 10176-2-AP, 1:2000, Proteintech, RRID:AB_2224574), rabbit anti-EGFR (Cat#: 18986-1-AP, 1:2000, Proteintech, RRID:AB_10596476), rabbit anti-EZH2 (Cat#: 5246S, 1: 1000, Cell Signaling Technology, RRID:AB_2714188), rabbit anti-H3 (Cat#: A2352, 1:1000, ABclonal, RRID:AB_2764312), rabbit anti-Vimentin (Cat#: ab92547, 1:1000, Abcam, RRID:AB_10562134), rabbit anti-H3K27me3 (Cat#: A2363, 1:1000, ABclonal, RRID:AB_2756439), mouse anti-γH2AX (Cat#: 80312, 1:1000, Cell Signaling Technology), rabbit anti-PARP1 (Cat#: 32138, 1:1000, Abcam, RRID:AB_777101), rabbit anti-BRCA1 (Cat#: ab245330, 1:1000, Abcam), rabbit anti-BARD1 (Cat#: 22964-1-AP, 1:1000, Proteintech, RRID:AB_2879190), rabbit anti-RAD51 (Cat#: ab133534, 1:1000, Abcam, RRID:AB_2722613), rabbit anti-53BP1 (Cat#: A3859, 1:1000, ABclonal), anti-BANF1 (Cat#: 30525-1-AP, 1:1000, Proteintech, RRID:AB_3086349), anti-NUMA1 (Cat#: 16607-1-AP, 1:1000, Proteintech, RRID:AB_2154616), rabbit anti-β-actin (Cat#: AC026, 1:1000, Abclonal, RRID:AB_2768234). Afterward, the blots were washed three times for 10 minutes each with TBST, incubated with secondary antibodies conjugated to horseradish peroxidase (HRP) for 1 h at room temperature, and washed again three times for 10 min TBST. The immunoblots were developed using Western ECL Substrate (Millipore, WBKLS0500).

### Karyotype analysis

Cells were synchronized at the G1/S phase boundary via colchicine (1.5 μg/ml) incubation for 25 min at 37 °C, followed by hypotonic treatment using sodium citrate (0.1 μg/ml) for 12 min before fixation with methanol-acetic acid (1:3). The cells were then spread onto slides and stained with Giemsa. Ploidy was assessed based on the definitions of the International System for Human Cytogenetic Nomenclature (ISCN).

### Neutral comet assays

The neutral comet assays were conducted using the Comet Assay Kit (Trevigen, 4250-050-K) following the manufacturer’s guidelines. Specifically, cells were exposed to the indicated compounds for 48 h, then harvested and washed twice with ice-cold PBS. Cell suspensions were then embedded in low-melting agarose and placed on comet slides. The slides were chilled at 4 °C for 30 min and treated with neutral lysis buffer overnight. Electrophoresis was then carried out at 21 V for 40 min. Afterward, the slides were immersed in 70% ethanol for 5 minutes, dried at 37 °C for 15 min, and stained with SYBR Gold nucleic acid gel stain (Invitrogen, S11494) for 30 min in the dark. Images were captured using a fluorescence microscope (Olympus CKX53). DNA damage was quantified by measuring the tail moment using the OpenComet Fiji plugin. At least 100 cells were analyzed for each condition.

### Immunofluorescence

The cells were cultured on glass coverslips (VWR, 631-0150), fixed with 4% (w/v) paraformaldehyde (Beyotime, P0099) for 10 minutes at room temperature, washed with PBS, permeabilized with 0.1% (v/v) Triton X-100 for 10 minutes, and then blocked with 1% BSA in PBS for 30 minutes. Subsequently, the cells were washed twice for 10 minutes with PBS and then incubated with the primary antibody diluted in PBS containing 1% BSA at 4 °C overnight. Primary antibody dilutions were as follows: mouse anti-γH2AX (Cat#: 80312, 1:400, Cell Signaling Technology, RRID:AB_2799949), rabbit anti-BRCA1 (Cat#: ab245330, 1:200, Abcam), rabbit anti-53BP1 (Cat#: A3859, 1:400, ABclonal). Afterward, the cells were washed twice with PBS and incubated with the appropriate secondary antibody for 1 h at room temperature. The corresponding secondary antibodies used were Alexa Fluor 488 goat anti-rabbit (Jackson, 611-544-215), Alexa Fluor 488 goat anti-rabbit (Invitrogen, A11034), and Alexa Fluor 594 goat anti-mouse (Jackson, 611-544-214). Cell nuclei were stained using DAPI (Beyotime, C1002).

Tissue tissue microarray immunofluorescence was conducted using TSA fluorescence triple staining kit (Cat#: RK05903, ABclonal). The primary antibody dilutions were anti-CBX2 (Cat#: MA5-38465, 1:200, ThermoFisher) and anti-EPCAM (Cat#: AB223582, 1:400, ABclonal). Cell nuclei were stained using DAPI (Beyotime, C1002).

### HR and NHEJ assays

To conduct integrated homologous recombination and non-homologous end-joining assays, the following plasmids were obtained from Addgene: pLCN DSB Repair Reporter (DRR) (Addgene plasmid #98895, RRID:Addgene_98895), pCAGGS DRR mCherry Donor EF1a BFP (Addgene plasmid #98896, RRID:Addgene_98896), and Isce1 (Addgene plasmid #26477, RRID:Addgene_26477). Stable OVCAR4-sgNC, OVCAR4-CBX2^KO^, OVCAR4-CBX2^IDRWT^, OVCAR4-CBX2^IDRMut^, CAOV3-sgNC, CAOV3-CBX2^KO^, CAOV3-CBX2^IDRWT^, CAOV3-CBX2^IDRMut^ cells containing the pLCN DSB Repair Reporter were generated through electroporation transfection (450Mv; 5 ms, Celetrix_LE). The stable cells were then transfected with Isce1 and an exogenous donor for HR (pCAGGS DRR mCherry Donor EF1a BFP). Forty-eight hours after transfection, cells were subjected to FACS analysis to detect BFP (as a control for transfection efficiency), GFP, or mCherry signals.

### Live cell imaging and FRAP

Indicated cells (2 × 10^5^) were seeded on a gelatin-coated 35-mm cover-glass bottom dish and incubated overnight_._ Cells were treated with indicated compounds and stained with HOECHST33342 for 30 min. Photobleaching was performed by using a Zeiss LSM 700 Observer with 1003/1.40 NA Oil-immersion Objective. The image size was 64.0 mm × 64.0 mm with an 8-bit image depth. For bleaching EGFP, 488 nm laser was used. The pinhole was fully open. Before photobleaching, two images were taken. After photobleaching, 60 images were taken with 3 s intervals. The images were analyzed by using Fiji. TurboReg was used to correct for translational movement. The fluorescence intensities were corrected for fluctuations and then normalized to the signal before bleaching.

### Chromatin-bound protein extraction

The chromatin-bound protein was extracted using the Subcellular Protein Fractionation Kit (ThermoFisher, 78840) according to the manufacturer’s instructions. The indicated cells were collected and washed with cold PBS. Following the protocol, cytoplastic extraction buffer was added to the cell pellet, and the tube was incubated at 4 °C for 10 min with gentle mixing. The mixture was then centrifuged at 500 × g for 5 min to obtain the supernatant (cytoplasmic extract). Next, membrane extraction buffer containing protease inhibitors was added, incubated at 4 °C for 10 min, and centrifuged at 3000 × g for 5 min to obtain the supernatant (membrane extract). Subsequently, nuclear extraction buffer was added to acquire the soluble nuclear extract. Finally, the chromatin-bound nuclear extract (CNE) was obtained using nuclear extraction buffer containing CaCl_2_ and cicrococcal nuclease.

### RNA sequencing

Total RNA was extracted and purified from OVCAR4-CBX2^NC^, OVCAR4-CBX2^IDRWT^, OVCAR4-CBX2^IDRMut^ and stored in TRIzol (Invitrogen, USA). Triplicate samples were harvested for each group. RNA-seq libraries were constructed using an Illumina stranded mRNA sample preparation kit (NEB, E7770) according to the manufacturer’s protocol and were sequenced on an Illumina NovaSeq 6000 sequencing machine with 150-base pair (bp) paired-end reads. Genes with log_2_ fold change ≥ 1 and adjusted *p*-value < 0.05 were counted as differentially expressed genes.

### Gene set enrichment analysis

GSEA was performed using GSEA software (http://www.gsea-msigdb. org/gsea/) with 1000 permutations. Gene sets used were obtained from MSigDB (Hallmark gene sets; reactome subset of canonical pathway from C2 databases). A custom HRD-associated genes were split into up-regulated and down-regulated in HRD as used as input into GSEA. *P*-values < 0.05 and false discovery rate (FDR) < 0.25 were used to select statistically significant gene sets.

### Proteomics

For chromatin binding proteomics, all indicated cells were treated with 15 μmol cisplatin for 18 h, followed by chromatin binding protein extraction. Chromatin binding proteins from OVCAR4-NC and OVCAR4-CBX2^KO^ were subjected to quantitative label-free nano LC-MS/MS analysis performed by Biotree Biomedical Technology Co., Ltd. (Shanghai, China) with a nano-UPLC (EASY-nLC1200) coupled to a Q Exactive HFX Orbitrap instrument (Thermo Fisher Scientific) with a nano-electrospray ion source. Raw MS files were processed using Proteome Discoverer (PD) software (Version 2.4.0.305) and the built-in Sequest HT search engine. Chromatin binding proteins from OVCAR4-CBX2^NC^, OVCAR4-CBX2^IDRWT^ and OVCAR4-CBX2^IDRMut^ were subjected to qualitative shotgun LC-MS/MS performed by Genechem (Shanghai, China) using a Q Exactive mass spectrometer coupled with an Easy nLC (Thermo Fisher Scientific, MA, USA). Raw data obtained was then imported into Proteome Discoverer 2.2 (Thermo Fisher Scientific) for protein identification, and then embedded Mascot 2.6 engines were used for database searches.

For CBX2 binding proteomics, CBX2-IDR^WT^ and CBX2-IDR^Mut^ binding protein lysates were obtained through immunoprecipitation, with IgG as control. Subsequent shotgun analyses were performed by Genechem (Shanghai, China) as mentioned above.

### Immunoprecipitation

The CBX2 knock-out OVCAR4 cells transfected with EGFP-tagged NC, CBX2-IDR^WT^, and CBX2-IDR^Mut^ were treated with 15 μmol cisplatin for 18 hours. After harvesting, the cells were lysed in 1 ml of IP-dedicated lysate. The lysates were centrifuged at 12,000 g for 15 min at 4 °C. A 100 μl volume of lysate was taken out as the Input, and the remaining lysates were incubated with CBX2 primary antibody rabbit anti-CBX2 (Cat#:703491, 1 μg/million cells, Invitrogen) overnight at 4 °C. Subsequently, magnetic beads were added and incubated for 3 h. After centrifugation, the supernatant was discarded, and the precipitate was washed with the cold lysate five times. Finally, the precipitation was either preserved for proteomic analysis or boiled in 1X loading buffer and subjected to western blot analysis as described.

### Drug Screening

For the preliminary screening, CBX2 knock-out OVCAR4 cells transfected with CBX2-IDR^WT^, and CBX2-IDR^Mut^ were seeded at a density of 5000 cells per well in a final volume of 100 μL per well of 96-well plates (Corning). After 24 h, cells were treated with the drug library with concentrations of 0.1 μM, 1 μM, and 10 μM for each indicated compound and 0.5% DMSO control for 72 h. Cell viability was measured using the CCK8 Cell Viability Assay (Promega) according to the manufacturer’s instructions and an Infinite M200 Pro microplate reader (Tecan). Area under the curve (AUC) values were calculated for each plot, and drugs were ranked based on the difference between the AUCs for OVCAR4 cells transfected with EGFP-tagged CBX2-IDR^WT^ and CBX2-IDR^Mut^. The differences between AUCs were calculated according to %AUC_Diff_ = ((AUC_IDR_^Mut^ - AUC_IDR_^WT^)/AUC_IDR_^Mut^)*100, with positive values indicating preferential activity against CBX2-IDR^WT^ cells. Top 14 compounds from the preliminary screening, together with cDDP and paclitaxel, were further assessed in a validation screening by a 6-point concentration of 0.01 μM, 0.1 μM, 1 μM, 5 μM, 10 μM, and 20 μM in triplicate. Compounds displaying >20%AUC_Diff_ values were then validated by at least three replicates of the MTT assay.

### In vivo tumor models

Subcutaneous tumor models were established to assess the drug responsiveness of cells with different CBX2 statuses to indicated compounds. Generally, CAOV3-NC, CAOV3-CBX2^KO^, CAOV3-CBX2^IDRWT^ and CAOV3-CBX2^IDRMut^ cells were injected subcutaneously (5 × 10^6^ cells) into female BALB/c nude mice. Mice bearing CAOV3-NC and CAOV3-CBX2^KO^ cells were randomly assigned to treatment with saline or cisplatin (3 mg/kg) every other day when tumor volume reached 50–100 mm^3^. Mice bearing CAOV3-CBX2^IDRWT^ and CAOV3-CBX2^IDRMut^ cells were randomly assigned to treatment of saline or cisplatin (3 mg/kg) every other day, solvent control or Niraparib, and DMSO or Ibrutinib. Tumor growth was monitored every 3 days by measurement of tumor diameters, and the tumor volume was calculated as follows: 0.5 × length × width^2^. At the end of treatment, all tumors were excised, weighed, and confirmed by histology.

### Organoid viability assay

The organoids were maintained in a 3D culture system and were seeded in low adherent 96-well plates, which were then treated with the indicated compounds for 72 h. Cell viability was determined by a CellTiter-Glo 3D Cell Viability Assay (Promega, G9683).

### Quantification and statistical analysis

IC50 values were determined using GraphPad Prism 9 software and a variable slope dose-response model via nonlinear regression. The statistical details of the experiments can be found in the figures and legends, and all experiments were repeated at least 3 times. The statistical analyses were performed using SPSS 27.0 software. All comparisons were performed in GraphPad Prism 9.0. (ns, not statistically significant, *p < 0.05, **p < 0.01, ***p < 0.001, and ****p < 0.0001).

Raw MS files were processed using Proteome Discoverer (PD) software (Version 2.4.0.305) and the built-in Sequest HT search engine. Chromatin bound proteins from CBX2 knock-out OVCAR4 cells transfected with EGFP-tagged NC, CBX2-IDR^WT^, and CBX2-IDR^Mut^ were subjected to qualitative shotgun LC-MS/MS performed by Genechem (Shanghai, China) using a Q Exactive mass spectrometer coupled with an Easy nLC (Thermo Fisher Scientific, MA, USA). Raw data obtained was then imported into Proteome Discoverer 2.2 (Thermo Fisher Scientific) for protein identification, and then embedded Mascot 2.6 engines were used for database searches.

## Supplementary information

Supporting Information

Table S1

Table S2

Table S3

Full Western Blots

## Data Availability

The data supporting the findings of this study are available from the corresponding author upon reasonable request.
