# Supplementary material for: CBX2 phase-separation contributes to homologous recombination repair and drug resistance in ovarian cancer
Source: Cell Death Dis. 2026 Mar 26;17(1):366. doi: 10.1038/s41419-026-08605-4 (PMC13039389; doi:10.1038/s41419-026-08605-4)
Supplement: Supplementary file 1 — Supporting Information [file 41419_2026_8605_MOESM1_ESM.docx]

Supporting Information

CBX2 phase-separation contributes to homologous recombination repair and drug resistance in ovarian cancer

Si Sun^1†^, Lin Huang^1†^, Yujia Ma^1,2†^, Zheng Wei^1,3^, Mengna Zhu^1,4^, Mengqing Chen^1^, Feiquan Ying^1^, Xiaoling Zhou^1,5^, Ping Yang^6,5^,Yiping Wen^1^, Qiang Yang^1^, Liqiong Cai^1^**^*^**, Yuan Zhang^1^**^*^**, Jing Cai^1^**^*^**

^1^Department of Obstetrics and Gynecology, Union Hospital, Tongji Medical College, Huazhong University of Science and Technology, Wuhan 430022, China.

^2^Department of Obstetrics and Gynecology, Peking University Third Hospital, Beijing, China.

^3^Department of Obstetrics and Gynecology, Third Hospital of Shanxi Medical University, Shanxi Bethune Hospital, Shanxi Academy of Medical Sciences, Tongji Shanxi Hospital, Taiyuan, China.

^4^Chinese Institutes for Medical Research, Capital Medical University, Beijing 100069, China.

^5^Department of Obstetrics and Gynecology, First Affiliated Hospital, School of Medicine, Shihezi University, Shihezi, China.

^6^Department of Obstetrics and Gynecology, Xinjiang Production and Construction Corps Hospital, Urumqi, 830002, China.

^†^These authors contributed equally to this work.

**^*^Corresponding author. Email:** jingcai@hust.edu.cn (J.C.), yuanzhang75@hust.edu.cn (Y.Z.), tjcailq@hust.edu.cn (L.C.).


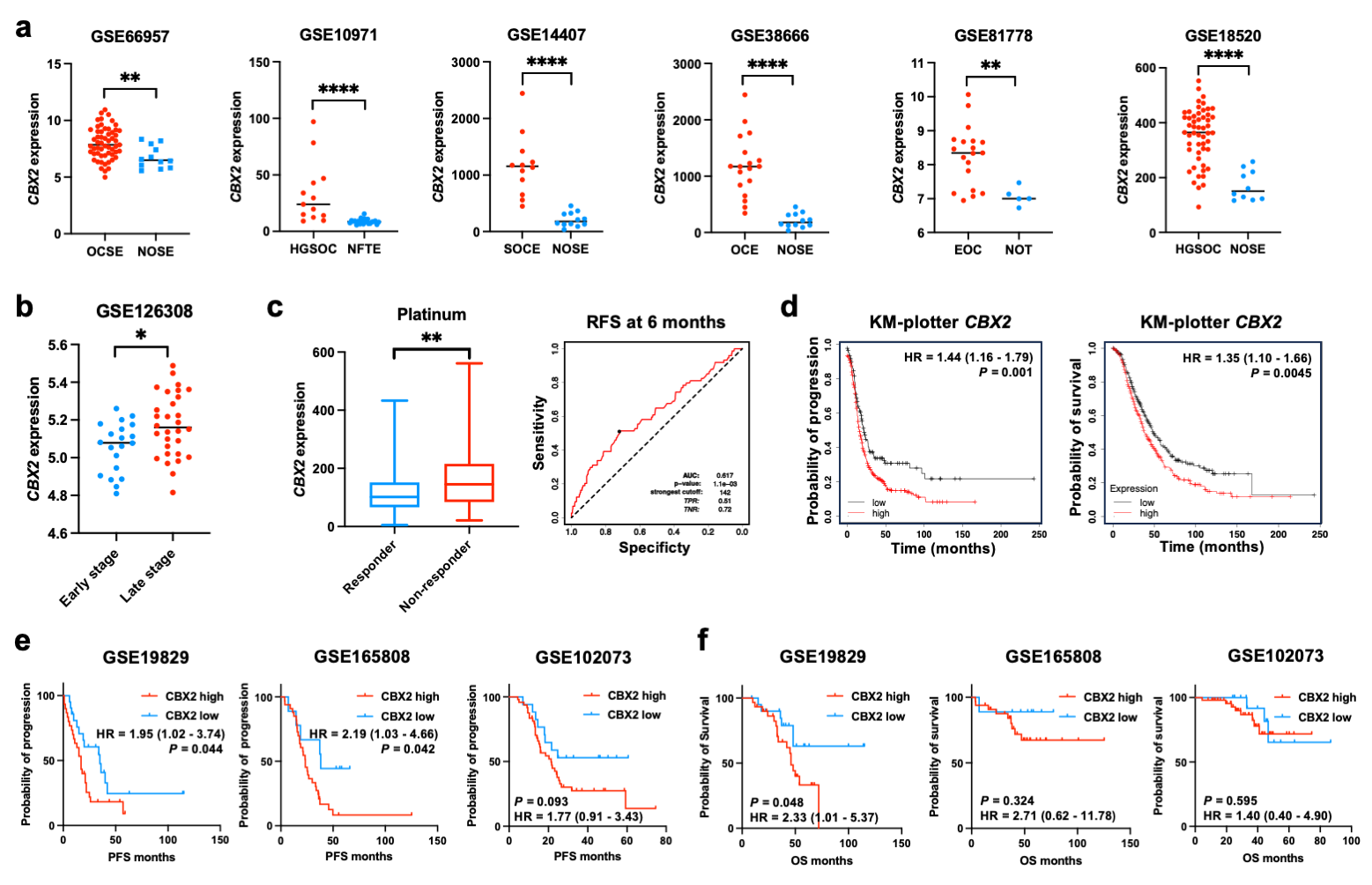


**Fig.S1. Overexpression of CBX2 in ovarian cancer tissues correlated with platinum resistance and poor survival of patients.** (a) *CBX2* expression in indicated ovarian cancer tissues and normal tissues based on data from GSE66957, GSE10971, GSE14407, GSE38666, GSE81778, and GSE18520 (unpaired two-tailed Student’s t test). (b) *CBX2* expression in early and late-stage ovarian cancer tissues based on data from GSE126308 (unpaired two-tailed Student’s t test). (c) Box plot and ROC curve of *CBX2* level in platinum responders, non-responders, and platinum response at 6 months (unpaired two-tailed Student’s t test). (d) Kaplan-Meier plot depicting progression-free survival and overall survival of ovarian cancer patients with high and low levels of *CBX2* using the KM plotter online database (log-rank test). (e) Kaplan-Meier plot depicting progression-free survival and (f) overall survival of ovarian cancer patients with high and low levels of *CBX2* using GEO online database GSE19829, GSE165808, and GSE102073. ns not significant, *P* > 0.05; * *P* < 0.05; ** *P* < 0.01; *** *P* < 0.001; **** *P* < 0.0001.


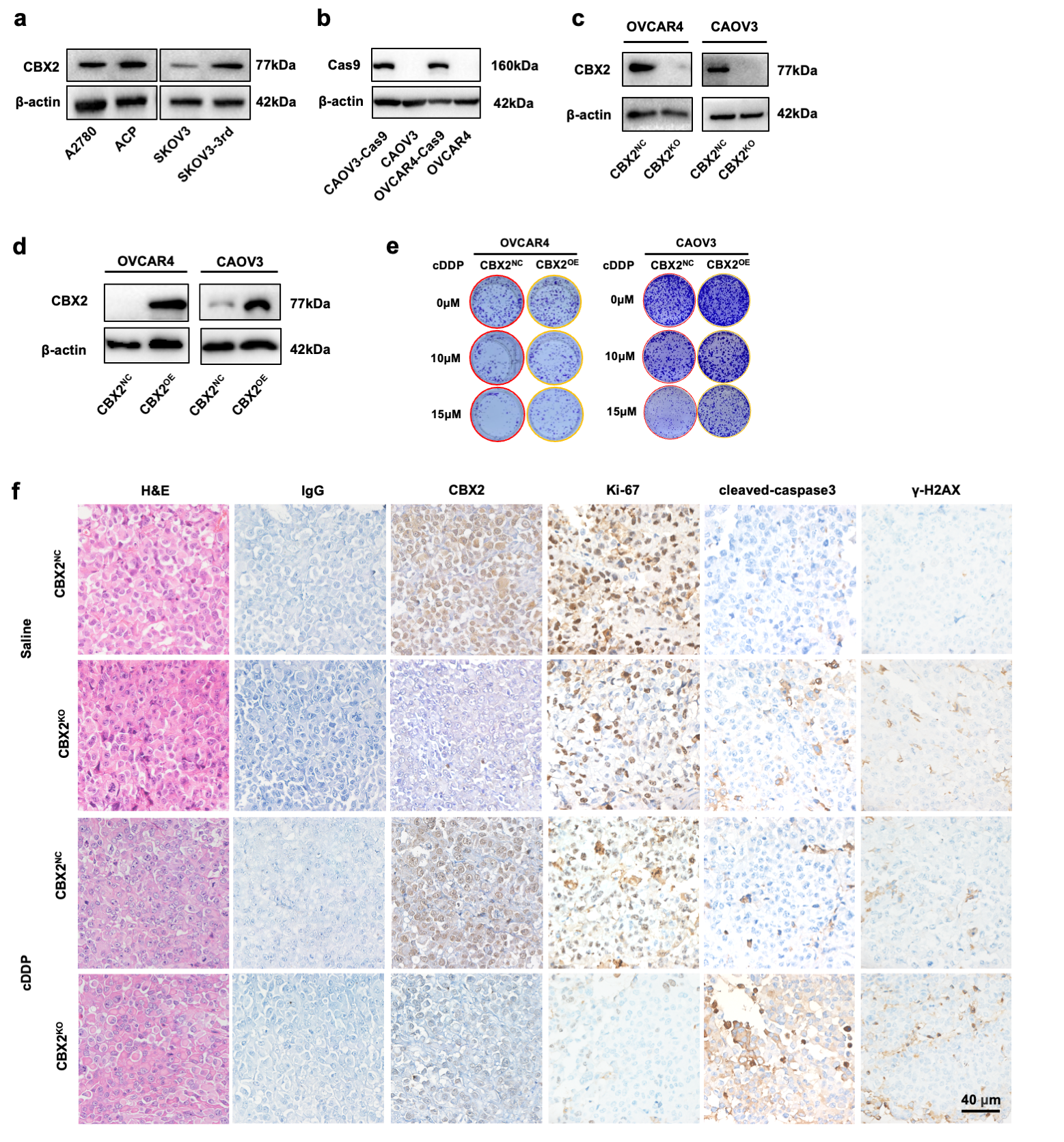


**Fig.S2.** **Related to Figure 1.** (a) Western blots of CBX2 protein levels in A2780, ACP, SKOV3, and SKOV3-3rd cells. (b) Western blots of Cas9 protein levels in OVCAR4 and CAOV3 cells. (c) Western blots of CBX2 protein levels of sgRNA targeted CBX2 (CBX2^KO^) or nontargeting control (CBX2^NC^) in OVCAR4 and CAOV3 cells. (d) Western blots of CBX2 protein levels in OVCAR4 and CAOV3 cells transfected with the CBX2 plasmid (CBX2^OE^) and control (CBX2^NC^). (e) Representative images of colony formation assay for CBX2-overexpressed OVRCA4 and CAOV3 cells compared to control cells. (f) Representative IHC images of CBX2, Ki67, cleaved caspase 3, and γH2AX staining in tumor xenograft tissues derived from CBX2-knockout and control cells. Scale bar, 40μm.


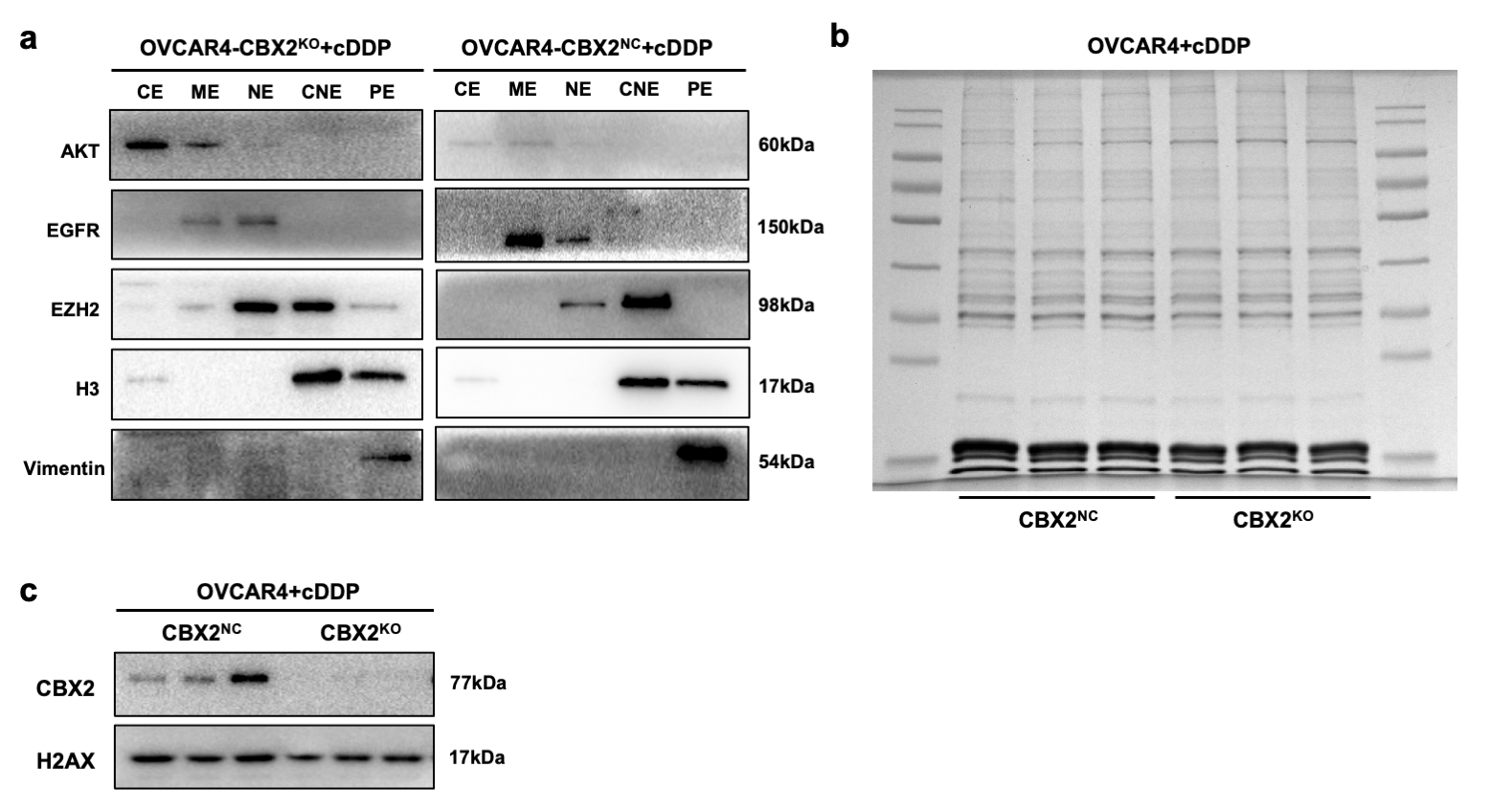


**Fig.S3. Related to Figure 2**. (a) Western blots of Akt, EGFR, EZH2, H3, and Vimentin of the indicated extracts of the CBX2 knockout (CBX2^KO^) and the control (CBX2^NC^) OVCAR4 cells treated with 15 μM cDDP for 18 hours. CE, cytoplasmic extract; ME, membrane extract; NE, nuclear extract; CNE, chromatin-bound nuclear extract; PE, pellet extract. (b) SDS page electrophoresis of the total protein of the CBX2^KO^ and the CBX2^NC^ OVCAR4 cells. (c) Western blot of CBX2 protein level in the CBX2^KO^ and the CBX2^NC^ OVCAR4 cells.


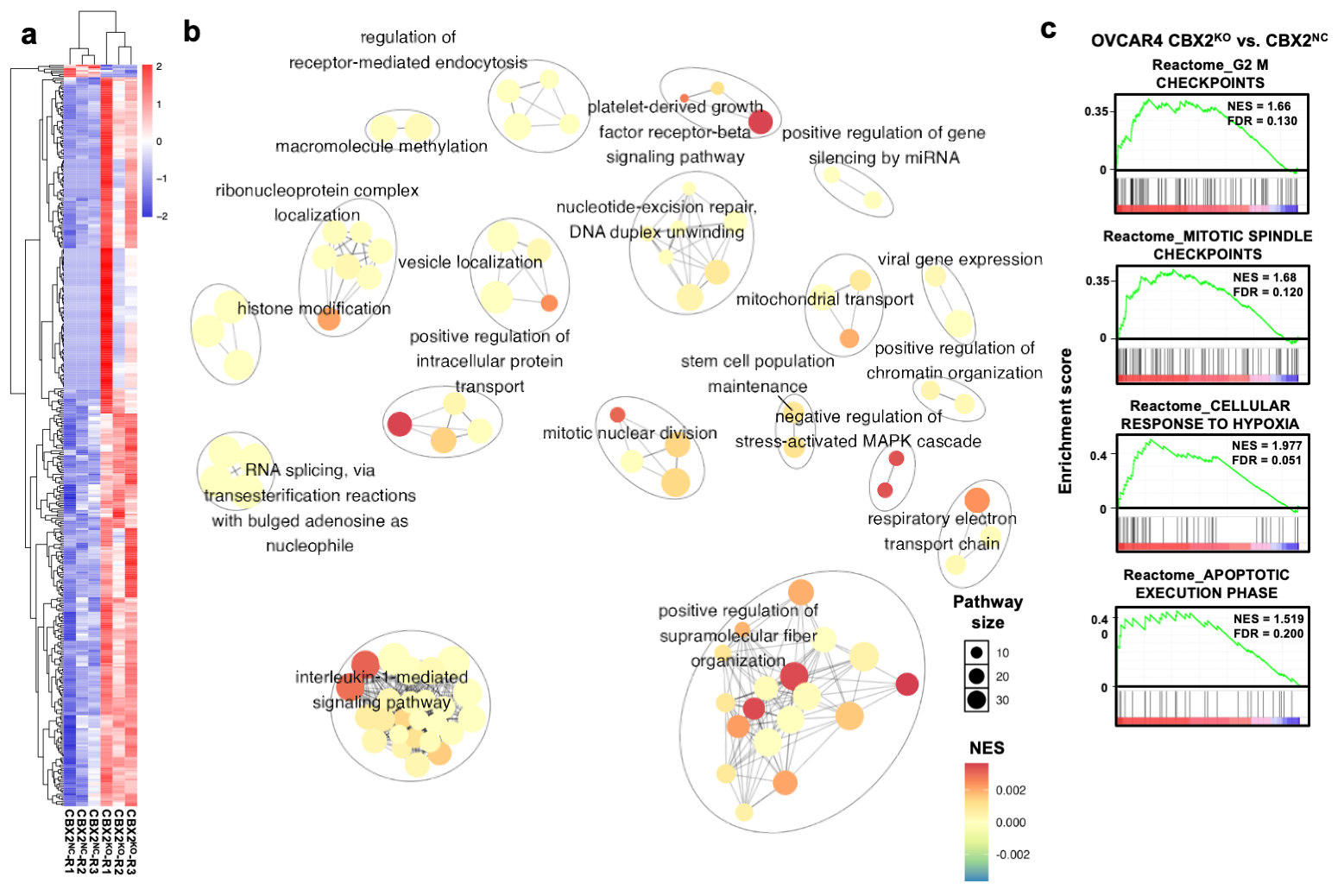


**Fig.S4. Related to Figure 2.** (a) Heatmap showing chromatin-bound protein changes between the CBX2 knockout (CBX2^KO^) and the control OVCAR4 cells. (b) GO-BP Enrichment network of the CBX2 knockout group compared to the control group, demonstrated using aPEAR. (c) Reactome GSEA enrichment score curves of chromatin-bound proteomics from CBX2-knockout cells (CBX2^KO^) compared to control cells (CBX2^NC^).


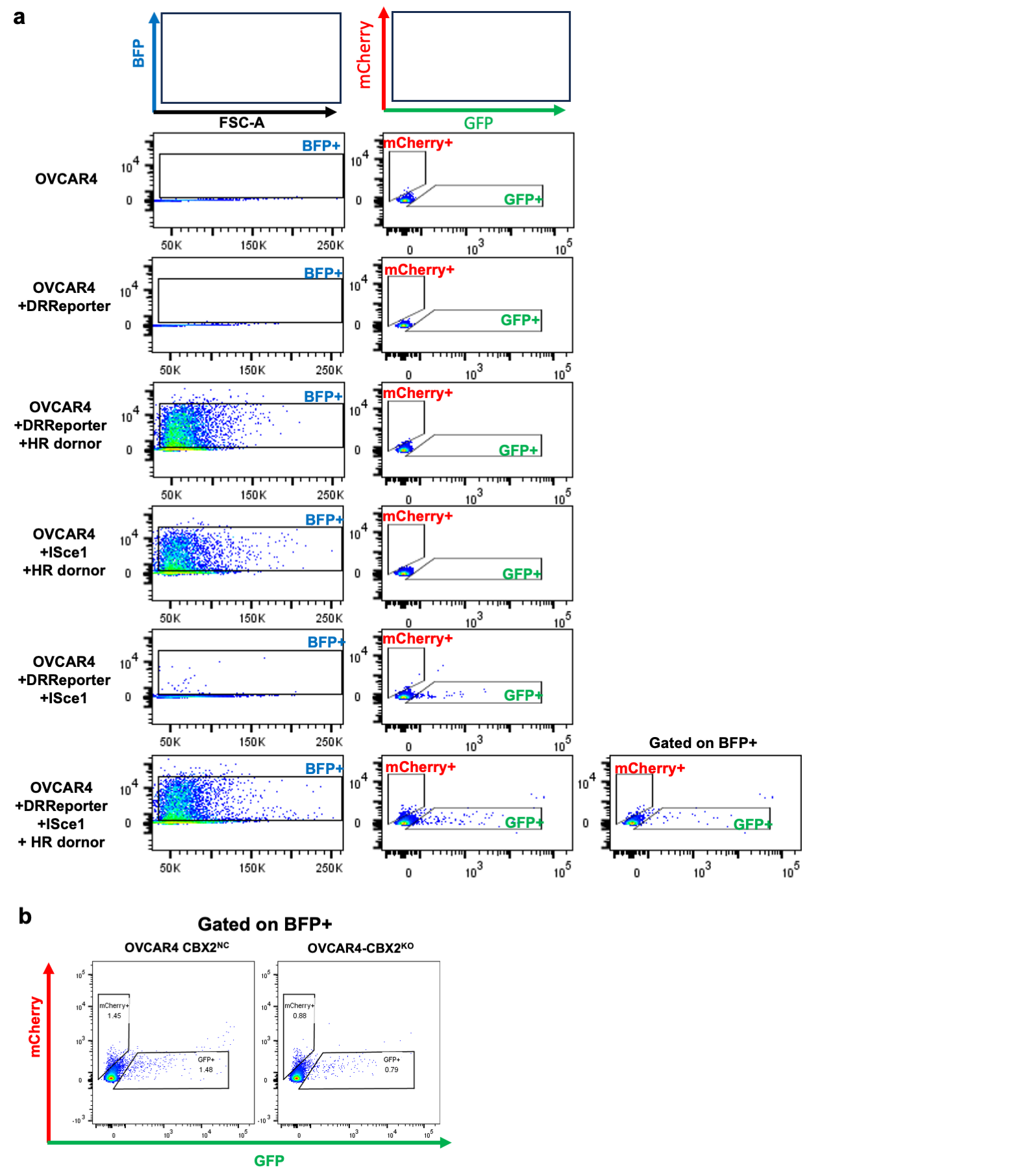


**Fig.S5. Related to Figure 2.** (a) Representative flow cytometry controls for the HR/NHEJ reporter assay. (b) Representative flow cytometry for HR/NHEJ repair efficiency in CBX2-knockout OVCAR4 cells and the control cells.

**
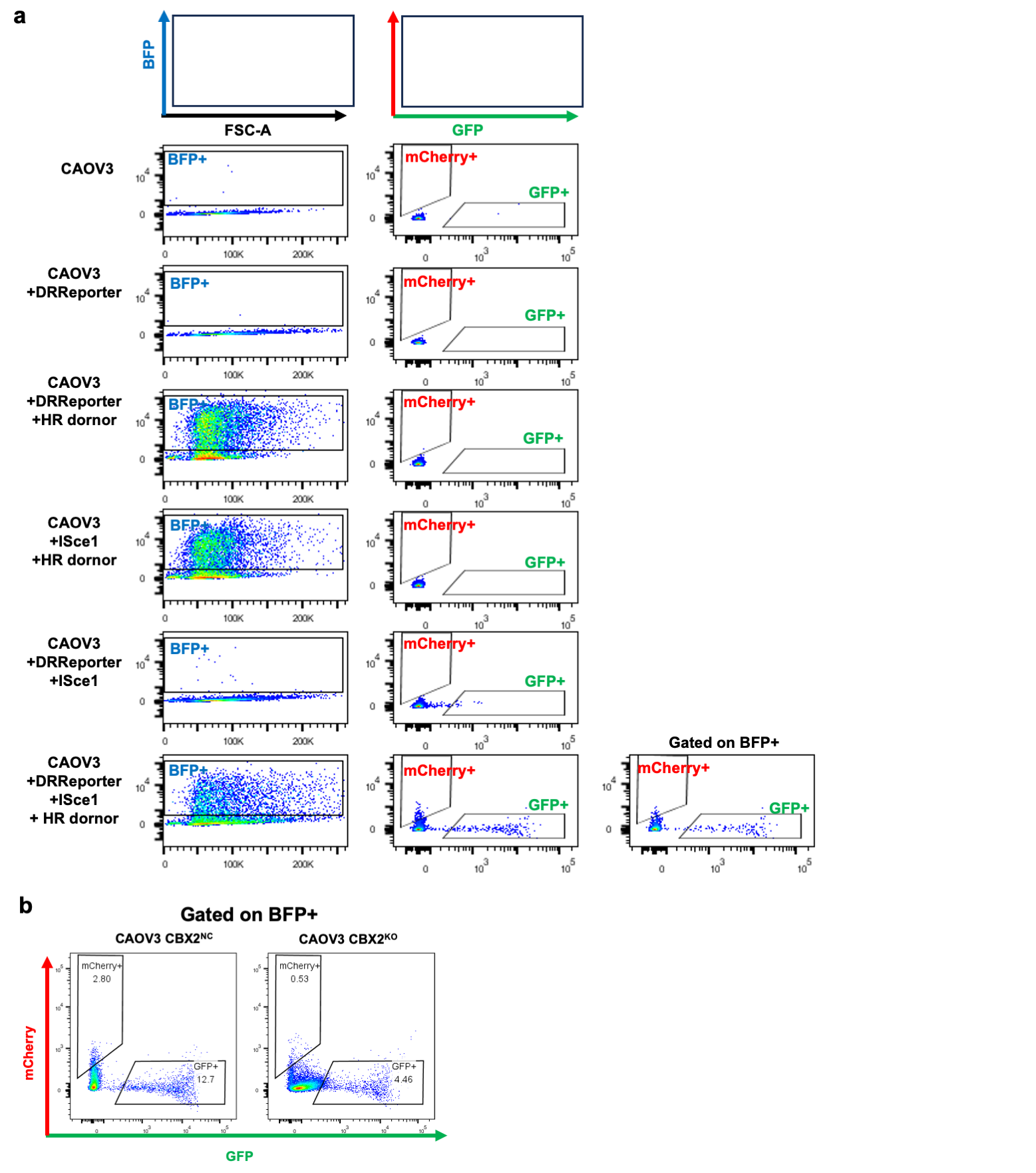
**

**Fig.S6. Related to Figure 2.** (a) Representative flow cytometry controls for the HR/NHEJ reporter assay. (b) Representative flow cytometry for HR/NHEJ repair efficiency in CBX2-knockout CAOV3 cells and the control cells.

**
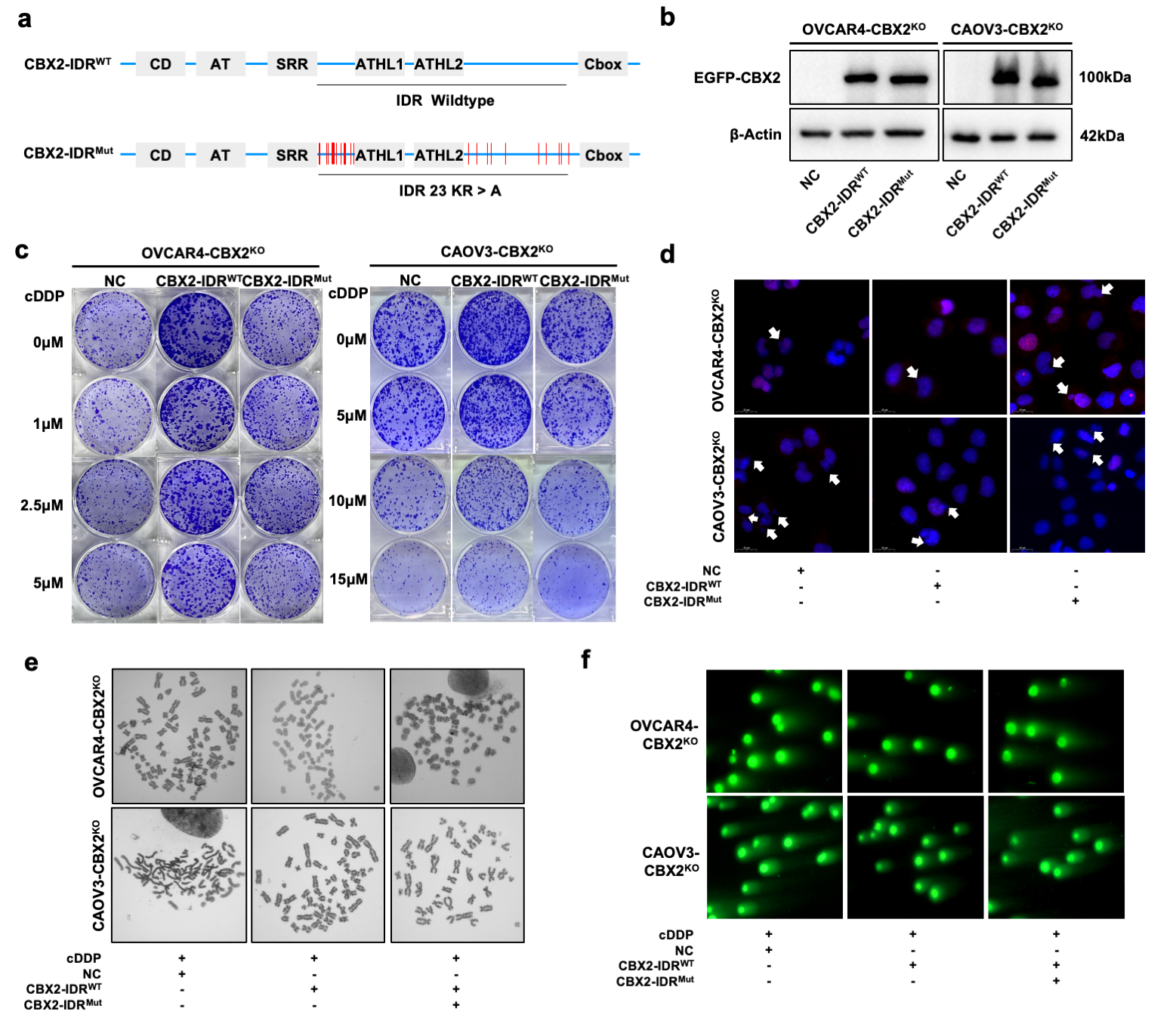
**

**Fig.S7. Related to Figure 3**. (a) Schematic illustration of CBX2-IDR^Mut^ and CBX2-IDR^WT^. (b) Western blots of CBX2 protein levels in the CBX2 knockout cells transfected with EGFP-tagged NC, CBX2-IDR^WT^, and CBX2-IDR^Mut^. (c) Representative images of colony formation assay for CBX2 knockout cells transfected with EGFP-tagged NC, CBX2-IDR^WT^, and CBX2-IDR^Mut^ after indicated cisplatin treatment for 14 days (unpaired two-tailed Student’s t test). (d) Representative images of γH2AX foci and micronuclei staining performed in CBX2 knockout cells transfected with EGFP-tagged NC, CBX2-IDR^WT^, and CBX2-IDR^Mut^. (e) Representative images of CBX2 knockout cells transfected with EGFP-tagged NC, CBX2-IDR^WT^, and CBX2-IDR^Mut^ at metaphase. (f) Representative images of the tail moment of the neutral comet assay of CBX2 knockout/knockdown cells transfected with EGFP-tagged NC, CBX2-IDR^WT^, and CBX2-IDR^Mut^ treated with 15 μM cisplatin and saline for 18 hours.


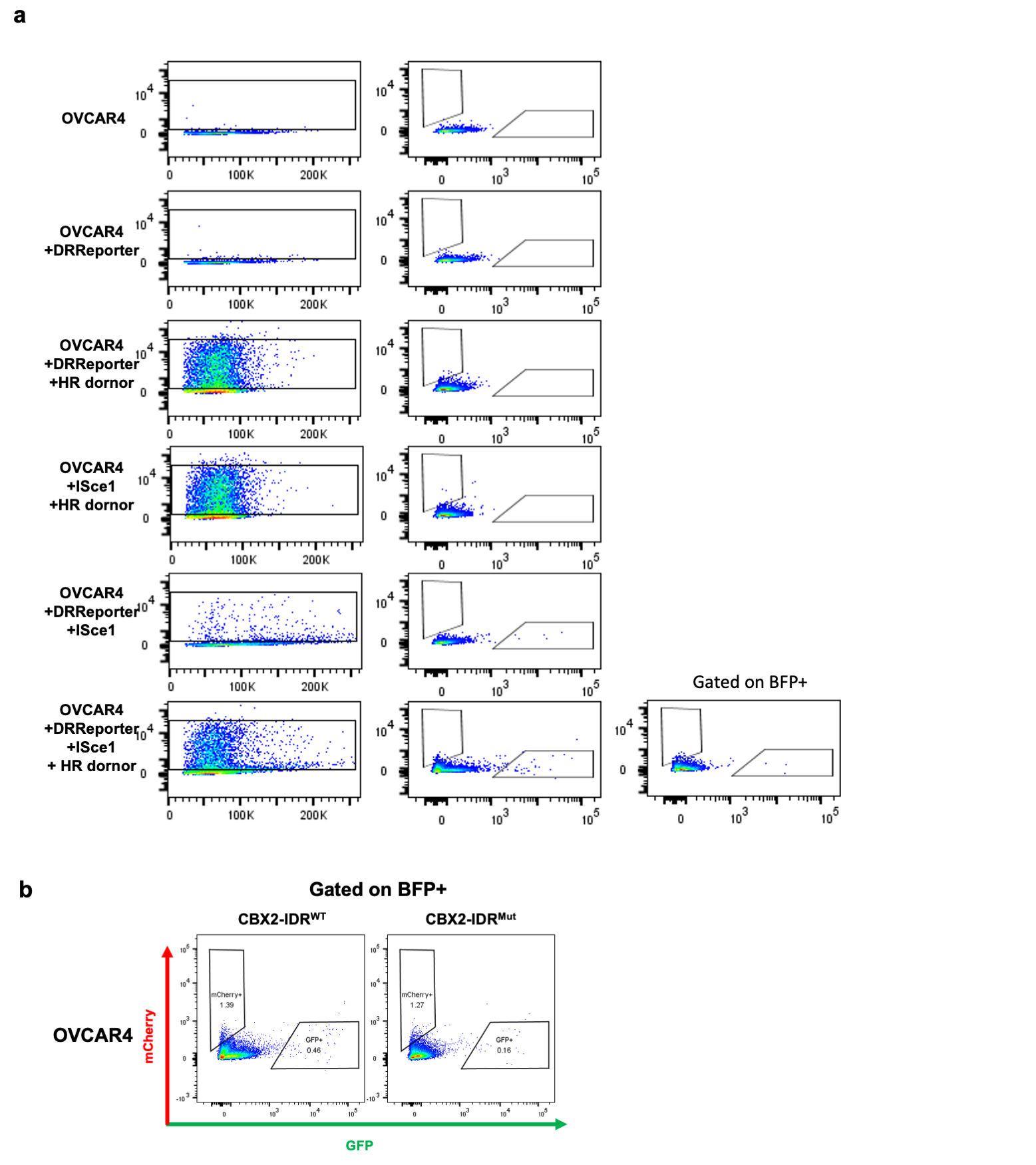


**Fig.S8. Related to Figure 3**. (a) Representative flow cytometry controls for the HR/NHEJ reporter assay. (b) Representative flow cytometry for HR/NHEJ repair efficiency in CBX2 knockout OVCAR4 cells transfected with EGFP-tagged CBX2-IDR^WT^ and CBX2-IDR^Mut^.


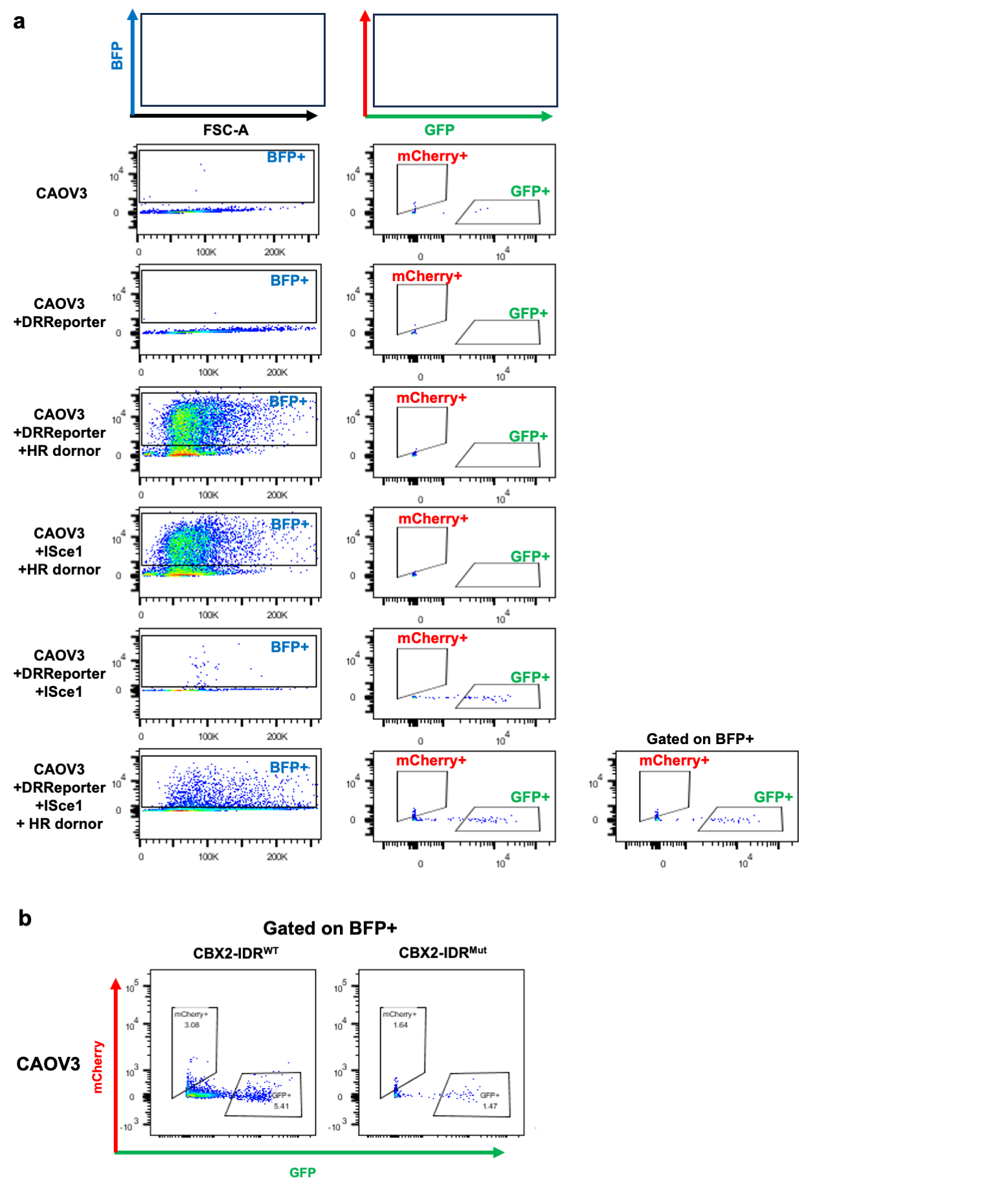


**Fig.S9. Related to Figure 3.** (a) Representative flow cytometry controls for the HR/NHEJ reporter assay. (b) Representative flow cytometry for HR/NHEJ repair efficiency in CBX2 knockout CAOV3 cells transfected with EGFP-tagged CBX2-IDR^WT^ and CBX2-IDR^Mut^.

**
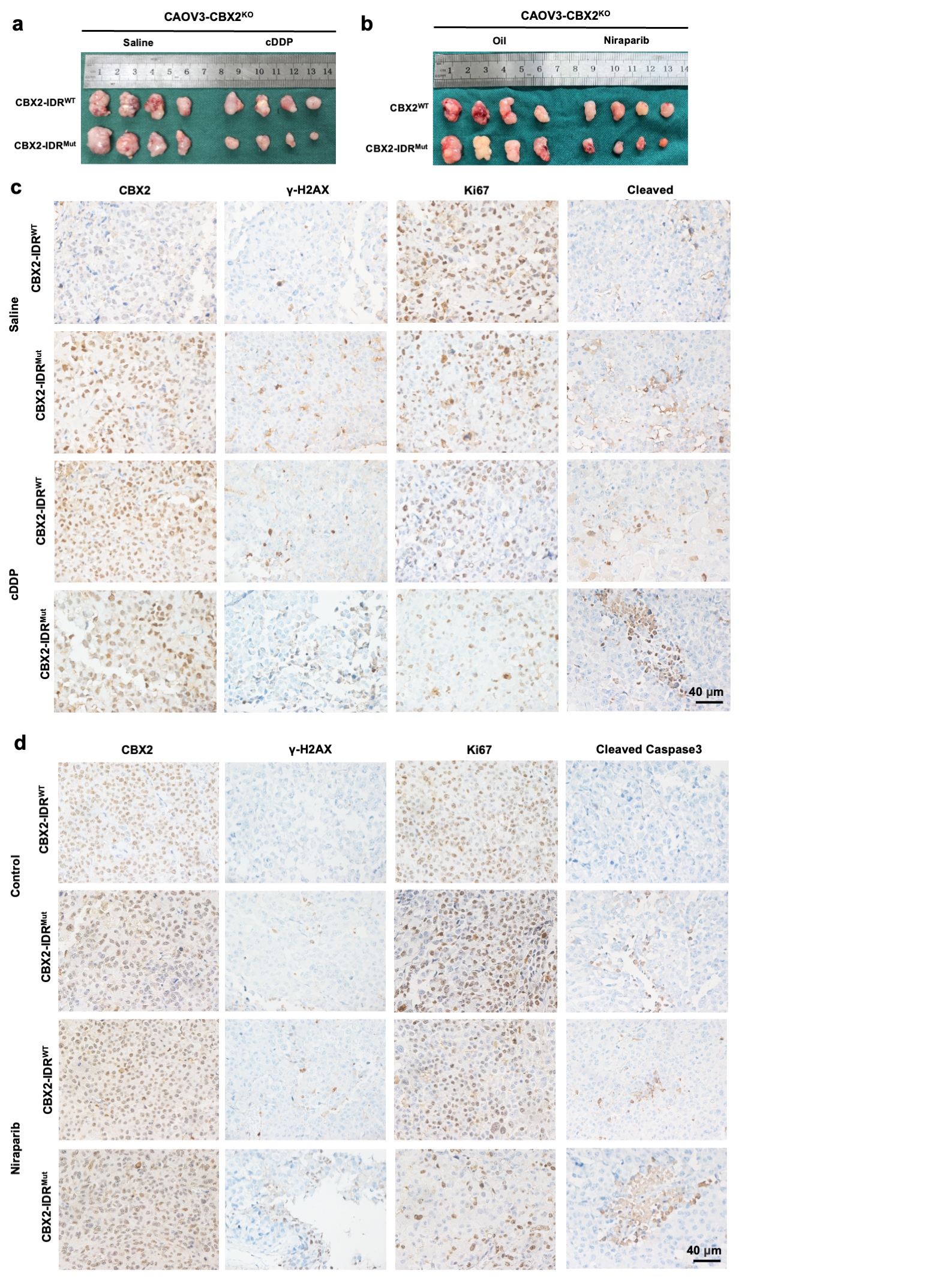
**

**Fig.S10. Related to Figure 3.** (a) Images of xenograft tumors derived from CBX2 knockout CAOV3 cells transfected with EGFP-tagged CBX2-IDR^WT^ and CBX2-IDR^Mut^ treated with cisplatin, and (b) Niraparib. (c) Representative IHC images of CBX2, Ki67, cleaved caspase 3, and γH2AX staining in tumor xenograft tissues derived from CBX2 knockout CAOV3 cells transfected with EGFP-tagged CBX2-IDR^WT^ and CBX2-IDR^Mut^ after exposure to cisplatin and (d) Niraparib.


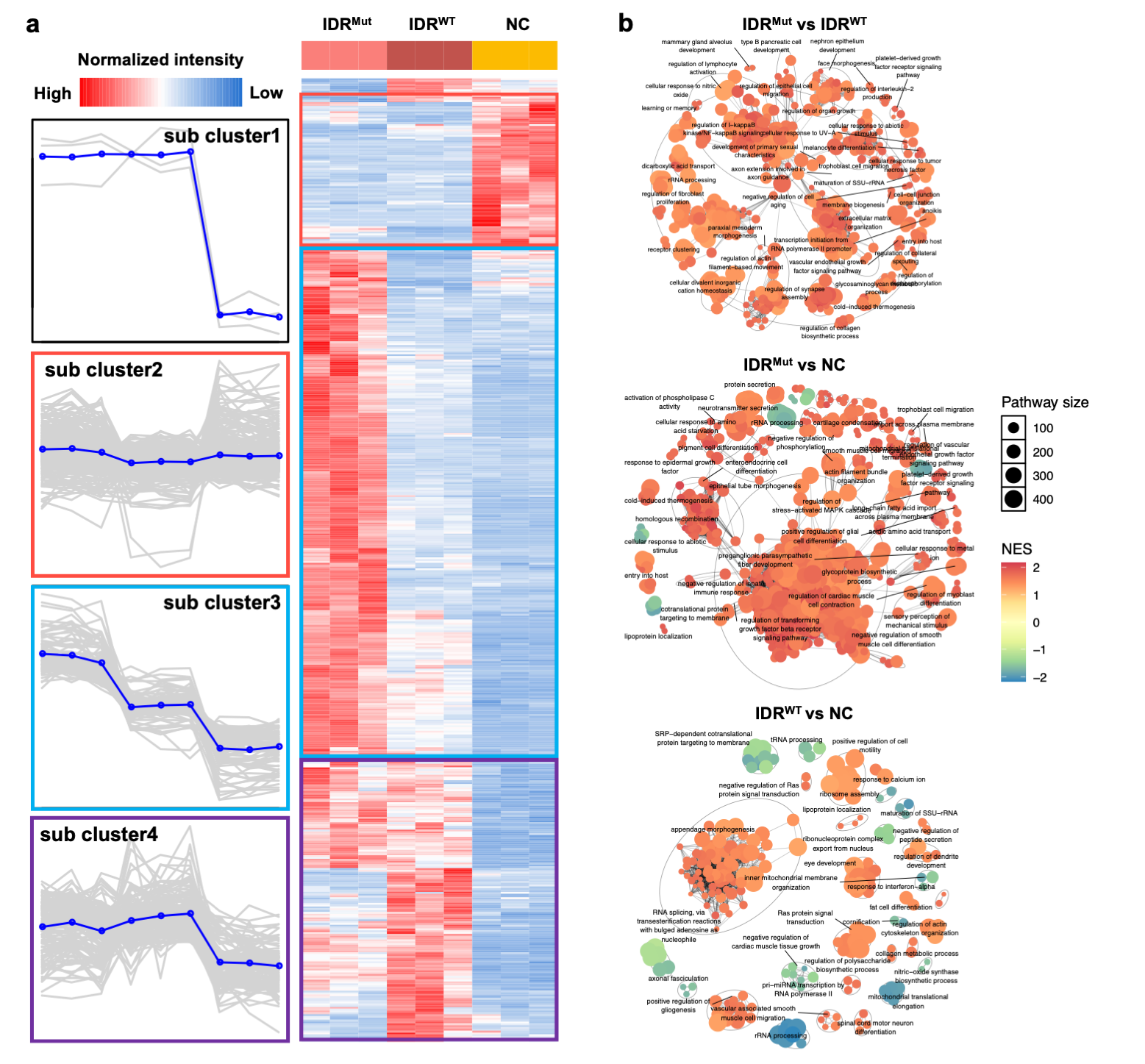


**Fig.S11. Related to Figure 4**. (a) Heatmap presenting sub-clusters of differentially expressed genes from the CBX2 knockout OVCAR4 cells transfected with EGFP-tagged CBX2-IDR^Mut^, CBX2-IDR^WT^, and the control groups. (b) GO-BP Enrichment network of CBX2 knockout OVCAR4 cells transfected with CBX2-IDR^Mut^, the CBX2-IDR^WT^, and the control groups demonstrated using aPEAR.


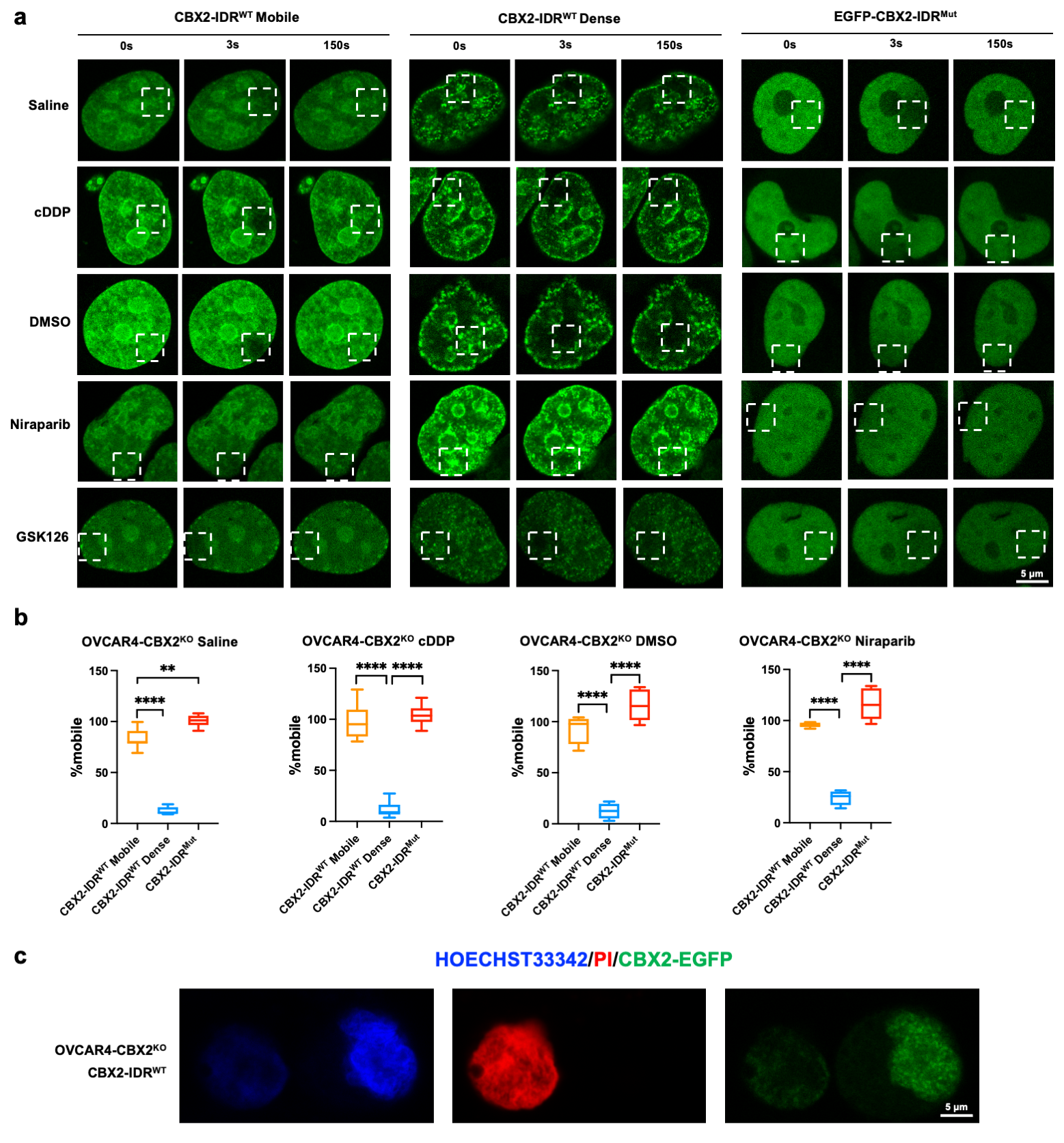


**Fig.S12. Related to Figure 5**. (a) Representative confocal images of FRAP of EGFP signal of EGFP-tagged mobile CBX2-IDR^WT^, dense CBX2-IDR^WT^, and CBX2-IDR^Mut^ in cells treated with saline, cDDP, DMSO, Niraparib, and GSK126 at pre-breaching, breaching, and post-breaching. Scale bar, 5μm. (b) Percentage of mobility of CBX2 in CBX2-IDR^Mut^ and mobile or dense CBX2-IDR^WT^ condensates. (c) Representative image of PI staining of cells with non-recovering dense EGFP-tagged CBX2-IDR^WT^ condensates. Scale bar, 5μm.


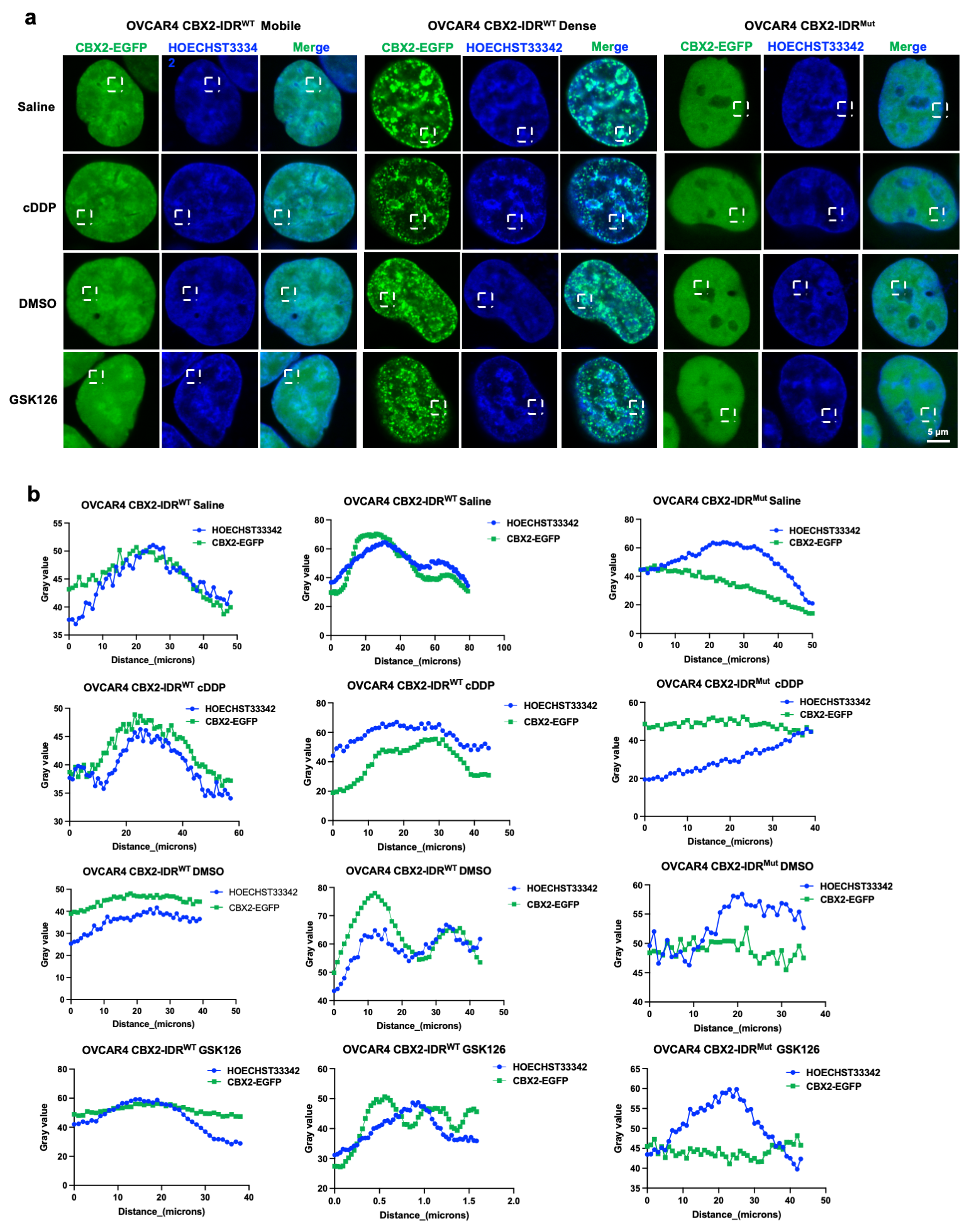


**Fig.S13. Mutation of the IDR of CBX2 alters the intranuclear distribution of CBX2.** (a) Representative images and (b) quantification of the co-localization of EGFP-tagged CBX2 and chromatin in CBX2 knockout OVCAR4 cells transfected with EGFP-tagged CBX2-IDR^WT^ and CBX2-IDR^Mut^, treated with the indicated compound. IDR, intrinsically disordered region.


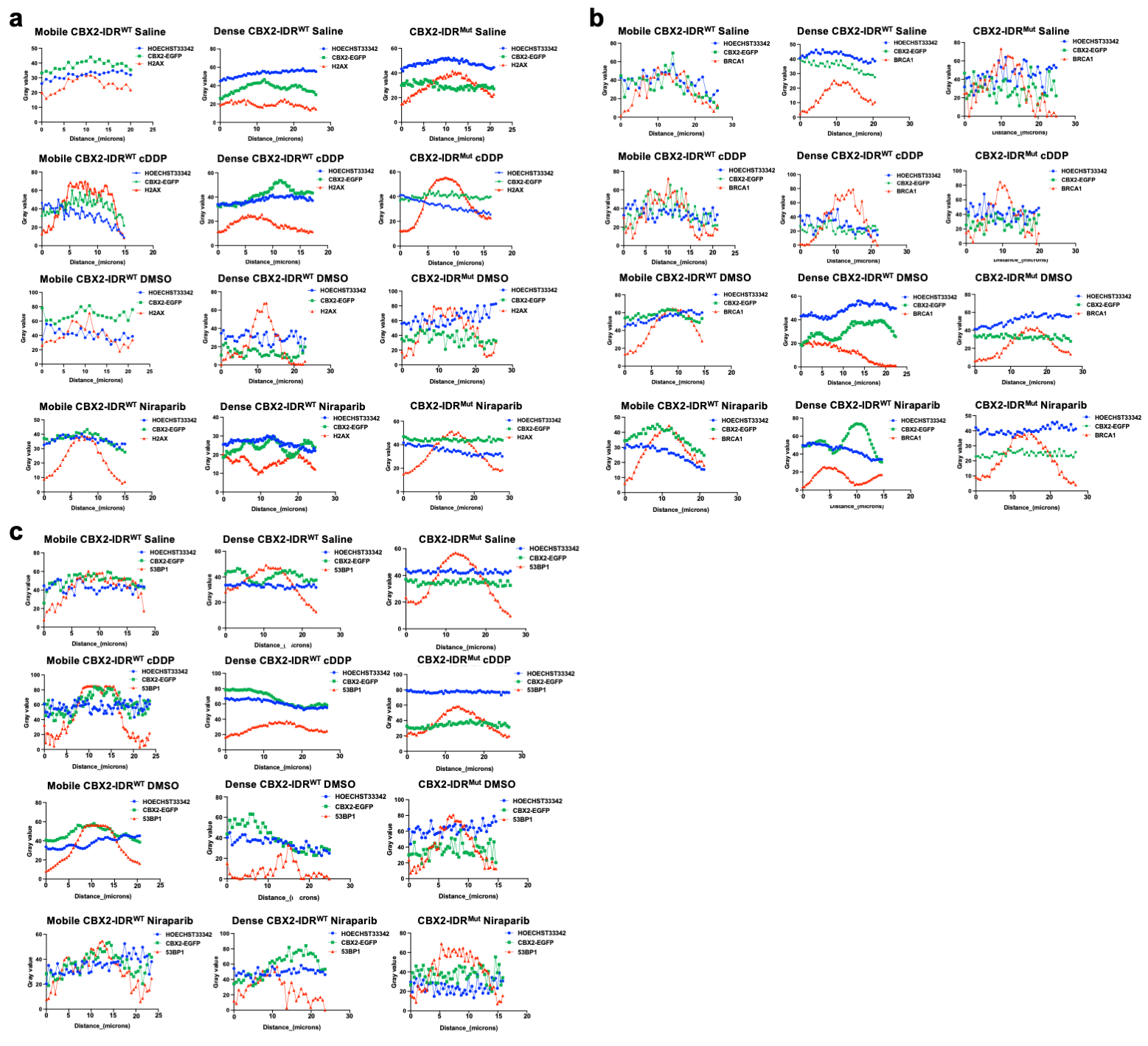


**Fig.S14. Co-localization data related to Fig. 5e.** (a) Quantification of the co-localization of EGFP-tagged CBX2 and γH2AX, (b) BRCA1, and (c) 53BP1 treated with the indicated compound. IDR, intrinsically disordered region.


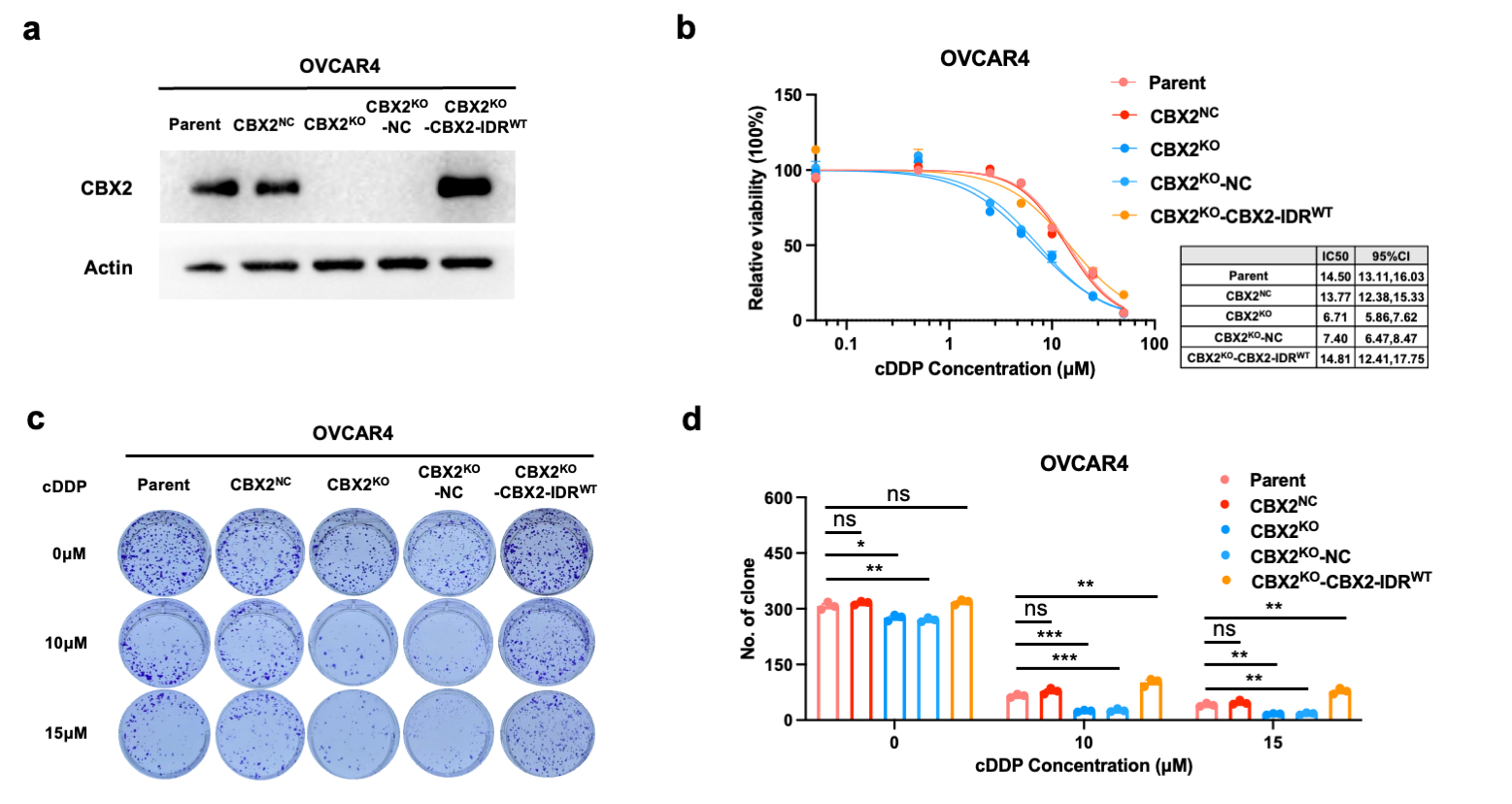


**Fig.S15. Homogeneous validation of EGFP-CBX2-IDR^WT^**. (a) Representative Western blots of the parental, CBX2^NC^, CBX2^KO^, CBX2^KO^-NC, and CBX2^KO^-CBX2-IDR^WT^ OVCAR4 cells. (b) Cell viability in the parental, CBX2^NC^, CBX2^KO^, CBX2^KO^-NC, and CBX2^KO^-CBX2-IDR^WT^ OVCAR4 cells after gradient concentrations of cDDP treatment for 72 hours detected by MTT assay. (c) Representative images and (d) quantification of colony formation assays for the parental, CBX2^NC^, CBX2^KO^, CBX2^KO^-NC, and CBX2^KO^-CBX2-IDR^WT^ OVCAR4 cells.


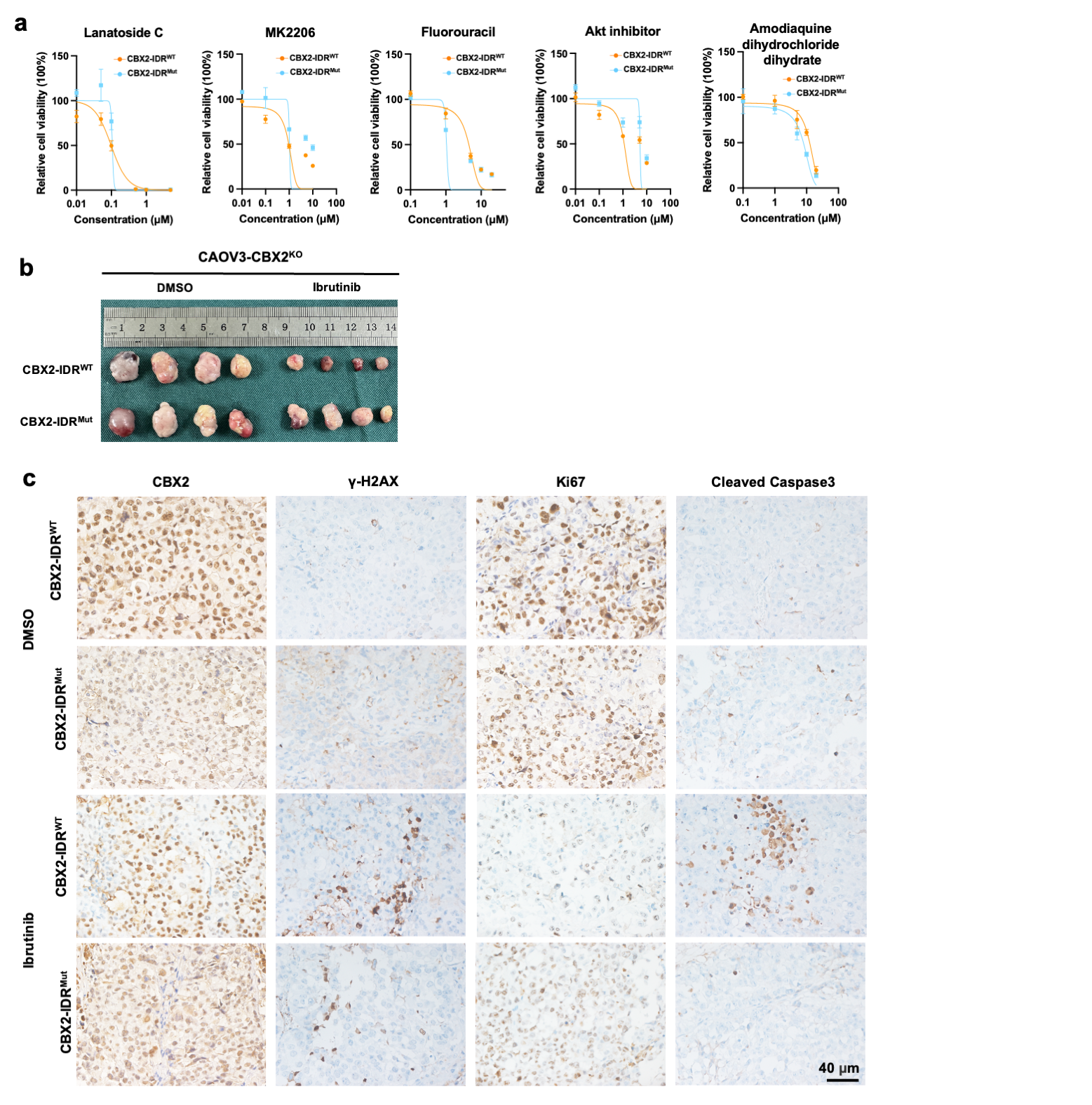


**Fig.S16. Related to Figure 6.** (a) Cell viability in the CBX2-IDR^WT^ and OVCAR4 cells after gradient concentrations of lanatoside C, MK2206, fluorouracil, Akt inhibitor, and amodiaquine dihydrochloride dihydrate treatment for 72 hours detected by MTT assay (b) Images of xenograft tumors derived from CBX2-knockout CAOV3 cells transfected with EGFP-tagged CBX2-IDR^WT^ and CBX2-IDR^Mut^, treated with Ibrutinib. (c) Representative IHC images of CBX2, Ki67, cleaved caspase 3, and γH2AX staining in tumor xenograft tissues derived from CBX2-knockout CAOV3 cells transfected with EGFP-tagged CBX2-IDR^WT^ and CBX2-IDR^Mut^ after exposure to Ibrutinib.


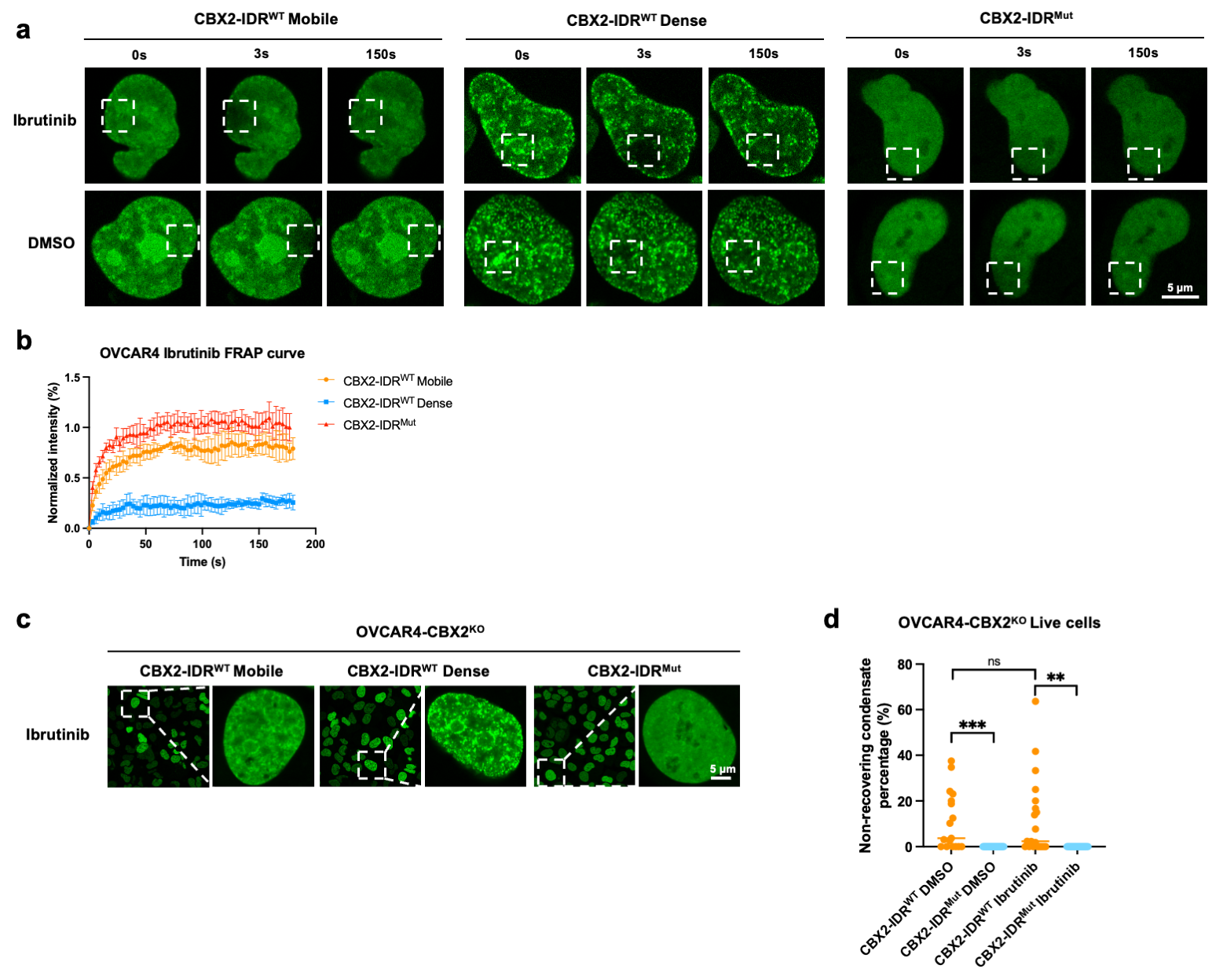


**Fig.S17. Ibrutinib did not alter the basic characteristics of CBX2 condensate.** (A) Representative confocal images of FRAP of EGFP signal of recovering mobile EGFP-tagged CBX2-IDR^WT^, non-recovering dense EGFP-tagged CBX2-IDR^WT^, and EGFP-tagged CBX2-IDR^Mut^ in CBX2-knockout OVCAR4 cells treated with Ibrutinib at pre-breaching, breaching, and post-breaching. Scale bar, 5μm. (B) Percentage of mobility of CBX2 in CBX2-knockout OVCAR4 cells transfected with CBX2-IDR^Mut^ and CBX2-IDR^WT^ condensates. (C) Representative images of EGFP-CBX2 distribution pattern in EGFP-tagged CBX2-IDR^Mut^ and CBX2-IDR^WT^ cells treated with Ibrutinib. Scale bar, 5 μm. (D) Quantification of the percentage of immobile dense CBX2 condensates in CBX2-knockout OVCAR4 cells transfected with CBX2-IDR^Mut^ and CBX2-IDR^WT^ treated with Ibrutinib. Data represent mean ± SEM of at least three independent biological replicates. ns not significant; ** *P* < 0.01; *** *P* < 0.001.


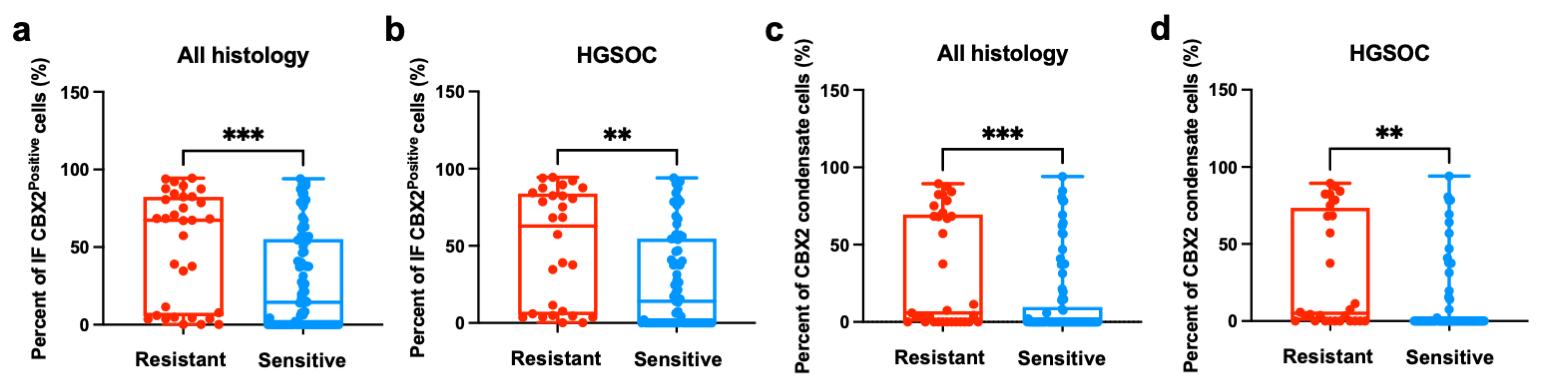


**Fig.S18. Fraction of cells with positive CBX2 signal and CBX2 condensate of tissue immunofluorescence assay.** (a) Box plots presenting the percent of positive CBX2 signal in the platinum-resistant and the platinum-sensitive groups from the ovarian cancer cohort with all histology and (b) HGSOC (Mann-Whitney test). (c) Box plots presenting the percent of cells with CBX2 condensate in the ovarian cancer cohort with all histology and (d) HGSOC (Mann-Whitney test). HGSOC, high-grade serous ovarian carcinoma. Data rep dresent mean ± SEM, ** *P* < 0.01; *** *P* < 0.001.
