# Supplementary material for: CBX2 phase-separation contributes to homologous recombination repair and drug resistance in ovarian cancer
Source: Cell Death Dis. 2026 Mar 26;17(1):366. doi: 10.1038/s41419-026-08605-4 (PMC13039389; doi:10.1038/s41419-026-08605-4)
Supplement: Supplementary file 2 — Table S1 [file 41419_2026_8605_MOESM2_ESM.docx]

Table S1. Clinical features and CBX2 IHC scores of 123 ovarian cancer patients and 101 high-grade serous ovarian carcinoma patients.

|  | Ovarian cancer | | | | HGSOC | | | |
| --- | --- | --- | --- | --- | --- | --- | --- | --- |
| Clinical features | No. of cases | CBX2 IHC score | | *P* | No. of cases | CBX2 IHC score | | *P* |
|  |  | High | Low |  |  | High | Low |  |
| Age at diagnosis (years) |  |  |  |  |  |  |  |  |
| <50 | 47 | 20 | 27 | 0.602 | 36 | 16 | 20 | 0.705 |
| ≥50 | 76 | 36 | 40 |  | 65 | 32 | 33 |  |
| Histology |  |  |  |  |  |  |  |  |
| High-grade serous | 101 | 48 | 53 | 0.340 | - | - | - | - |
| Others | 22 | 8 | 14 |  | - | - | - | - |
| FIGO stage |  |  |  |  |  |  |  |  |
| I-II | 29 | 8 | 21 | **0.026** | 25 | 7 | 18 | **0.024** |
| III-IV | 94 | 48 | 46 |  | 76 | 41 | 35 |  |
| Chemotherapy response |  |  |  |  |  |  |  |  |
| Platinum sensitive | 90 | 28 | 62 | **<0.001** | 73 | 23 | 50 | **<0.001** |
| Platinum resistance | 33 | 28 | 5 |  | 28 | 25 | 3 |  |

IHC, immunohistochemistry; IF, immunofluorescence; HGSOC, high-grade serous ovarian carcinoma. Chi-square and Fisher’s exact tests.
