# Supplementary material for: CBX2 phase-separation contributes to homologous recombination repair and drug resistance in ovarian cancer
Source: Cell Death Dis. 2026 Mar 26;17(1):366. doi: 10.1038/s41419-026-08605-4 (PMC13039389; doi:10.1038/s41419-026-08605-4)
Supplement: Supplementary file 3 — Table S2 [file 41419_2026_8605_MOESM3_ESM.docx]

Table S2. Clinical features and immunofluorescence statuses of CBX2 in 123 ovarian cancer patients.

| Clinical features | No. of cases | CBX2 IF signal | |  | CBX2 IF pattern | | |  | CBX2 IF pattern | |  |
| --- | --- | --- | --- | --- | --- | --- | --- | --- | --- | --- | --- |
|  |  | Positive | Negative | *P* | Non-condensate | Condensate | Negative | *P* | Condensate | Non-condensate and Negative | *P* |
| Age at diagnosis (years) |  |  |  |  |  |  |  |  |  |  |  |
| <50 | 47 | 34 | 13 | 0.364 | 15 | 19 | 13 | 0.661 | 19 | 28 | 0.691 |
| ≥50 | 76 | 49 | 27 |  | 21 | 28 | 27 |  | 28 | 48 |  |
| Histology |  |  |  |  |  |  |  |  |  |  |  |
| High-grade serous | 101 | 68 | 33 | 0.940 | 32 | 36 | 33 | 0.350 | 36 | 65 | 0.213 |
| Others | 22 | 15 | 7 |  | 4 | 11 | 7 |  | 11 | 11 |  |
| FIGO stage |  |  |  |  |  |  |  |  |  |  |  |
| I-II | 34 | 20 | 14 | 0.206 | 9 | 11 | 14 | 0.443 | 11 | 23 | 0.407 |
| III-IV | 89 | 63 | 26 |  | 27 | 36 | 26 |  | 36 | 53 |  |
| Chemotherapy response |  |  |  |  |  |  |  |  |  |  |  |
| Platinum sensitive | 90 | 53 | 37 | **<0.001** | 27 | 26 | 37 | **<0.001** | 26 | 64 | **<0.001** |
| Platinum resistance | 33 | 30 | 3 |  | 9 | 21 | 3 |  | 21 | 12 |  |

IF, immunofluorescence. Chi-square, Fisher’s exact, and Kruskal- Wallis tests.
