# Supplementary material for: CBX2 phase-separation contributes to homologous recombination repair and drug resistance in ovarian cancer
Source: Cell Death Dis. 2026 Mar 26;17(1):366. doi: 10.1038/s41419-026-08605-4 (PMC13039389; doi:10.1038/s41419-026-08605-4)
Supplement: Supplementary file 4 — Table S3 [file 41419_2026_8605_MOESM4_ESM.docx]

Table S3. Clinical features and immunofluorescence statuses of CBX2 in 101 high-grade serous ovarian carcinoma patients.

| Clinical features | No. of cases | CBX2 IF signal | |  | CBX2 IF pattern | | |  | CBX2 IF pattern | |  |
| --- | --- | --- | --- | --- | --- | --- | --- | --- | --- | --- | --- |
|  |  | Positive | Negative | *P* | Non-condensate | Condensate | Negative | *P* | Condensate | Non-condensate and Negative | *P* |
| Age at diagnosis (years) |  |  |  |  |  |  |  |  |  |  |  |
| <50 | 36 | 23 | 13 | 0.819 | 12 | 13 | 11 | 0.939 | 13 | 23 | 0.941 |
| ≥50 | 65 | 43 | 22 |  | 20 | 23 | 22 |  | 23 | 42 |  |
| FIGO stage |  |  |  |  |  |  |  |  |  |  |  |
| I-II | 25 | 12 | 13 | **0.018** | 6 | 6 | 13 | 0.0586 | 6 | 19 | 0.161 |
| III-IV | 76 | 56 | 20 |  | 26 | 30 | 20 |  | 30 | 46 |  |
| Chemotherapy response |  |  |  |  |  |  |  |  |  |  |  |
| Platinum sensitive | 73 | 42 | 31 | **<0.001** | 24 | 18 | 31 | **<0.001** | 18 | 55 | **<0.001** |
| Platinum resistance | 28 | 26 | 2 |  | 8 | 18 | 2 |  | 18 | 10 |  |

IF, immunofluorescence. Chi-square, Fisher’s exact, and Kruskal- Wallis tests.
