# Supplementary figures and images for: CBX2 phase-separation contributes to homologous recombination repair and drug resistance in ovarian cancer
Source: Cell Death Dis. 2026 Mar 26;17(1):366. doi: 10.1038/s41419-026-08605-4 (PMC13039389; doi:10.1038/s41419-026-08605-4)

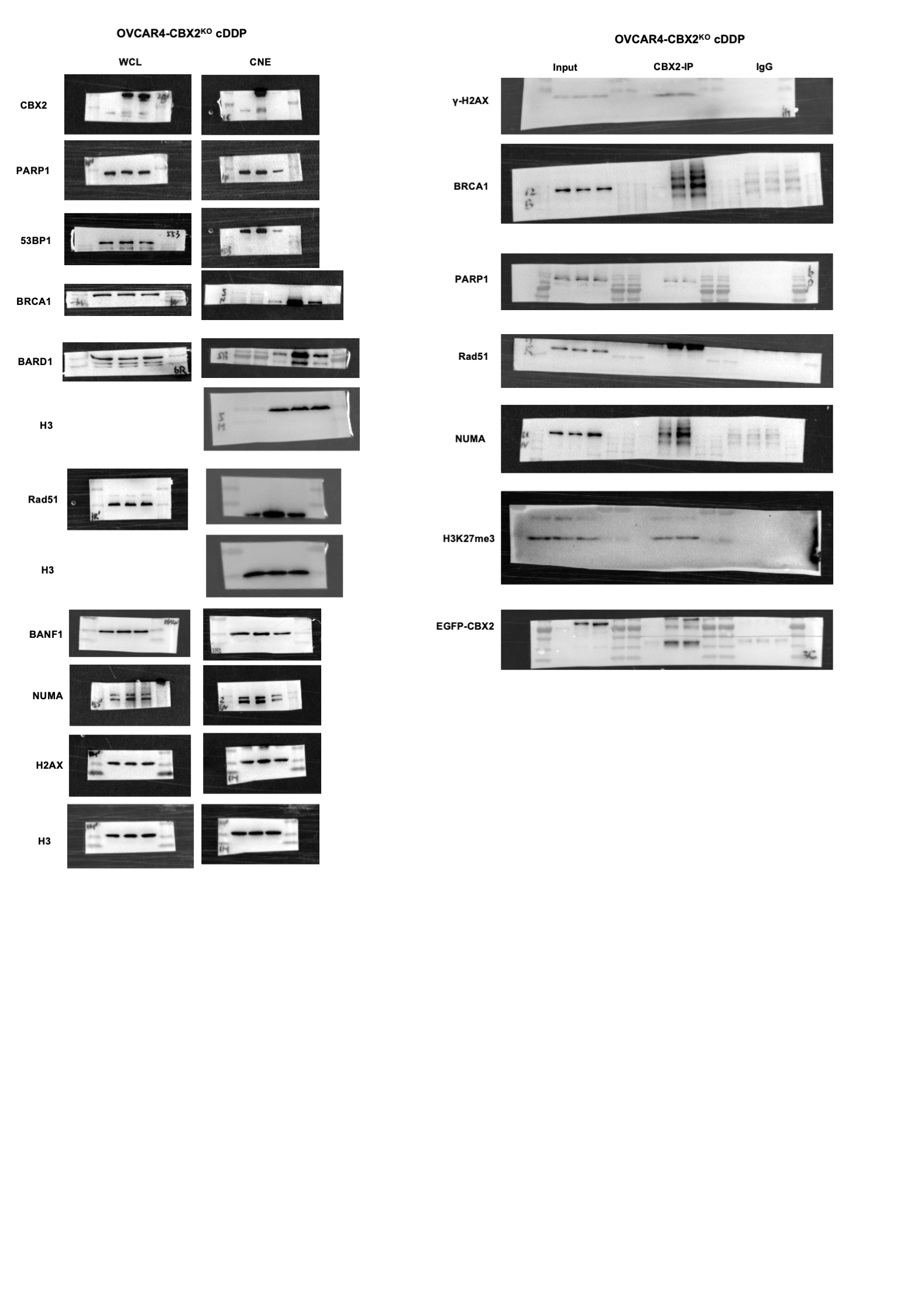

Supplement: Supplementary file 5 — Full Western Blots [file 41419_2026_8605_MOESM5_ESM.tif]
